# CRISPR-enabled biosensing of cancer-associated DNA methylation in cell-free DNA: molecular architectures, molecular sampling constraints, and translational biosensor engineering

**DOI:** 10.3389/fbioe.2026.1986098

**Published:** 2026-10-27

**Authors:** Hongzhi Luo, Xiaoxue Huang, Qiujie Yu, Qianyi Li, He Zha

**Affiliations:** 1 West China Hospital Sichuan University Jintang Hospital, Jintang First People’s Hospital, Chengdu, China; 2 Department of Laboratory Medicine, The Third Affiliated Hospital of Zunyi Medical University (The First People’s Hospital of Zunyi), Zunyi, Guizhou, China; 3 The Second Affiliated Hospital of Guizhou, University of Traditional Chinese Medicine, Guiyang, China; 4 Public Health Clinical Center of Chengdu, Chengdu, China

**Keywords:** bioelectronics, cancer diagnostics, cell-free DNA, CRISPR biosensors, DNA methylation, liquid biopsy, molecular residual disease, point-of-care diagnostics

## Abstract

DNA methylation in circulating cell-free DNA (cfDNA) provides cancer-associated and tissue-of-origin information complementary to mutation-based liquid biopsy. Clustered regularly interspaced short palindromic repeats (CRISPR)-based sensing is challenging because 5-methylcytosine (5 mC) generally preserves Watson-Crick base pairing and is not inherently resolved by sequence-programmed CRISPR-associated (Cas) proteins. This targeted narrative review examines how methylated and unmethylated molecules are distinguished and converted into Cas-readable outputs under fragmented, low-input cfDNA conditions. We organize the literature into four discrimination routes comprising sequence conversion, enzymatic gating, affinity-assisted recognition, and native-state Cas modulation. Engineered molecular transduction is considered separately as a downstream enabling layer. We compare readouts in terms of quantitative fidelity and integration requirements. Sequence-conversion and enzymatic-gating systems have the broadest analytical evidence among the identified studies, largely from *in vitro* experiments. Direct affinity-assisted 5 mC-to-Cas sensing remains sparsely studied, and evidence for native-state sensing in patient-derived cfDNA is limited to exploratory observational comparisons. No native-state Cas methylation assay identified in this review has established clinically usable diagnostic performance. Reported analytical limits of detection and methylation-fraction discrimination cannot be extrapolated to clinical sensitivity without accounting for input, recovery, fragmentation, background, and decision thresholds. Translation requires validated biomarkers, fragment-matched calibration, orthogonal reference testing, reproducible device integration, and independent evaluation of locked assays in intended-use populations.

## 1 Introduction

Circulating cell-free DNA (cfDNA) is an important liquid-biopsy analyte because plasma contains short DNA fragments released from multiple tissues, including malignant clones. The amount of circulating tumor DNA (ctDNA) available for analysis is determined by tumor biology, shedding, plasma volume, and molecular recovery. Analytical sensitivity cannot compensate for an aliquot that contains no informative tumor-derived molecules. Mutation-based clinical studies reported associations between ctDNA detectability, cancer type, and disease stage (Bettegowda et al., 2014). Later reviews and implementation studies highlighted the additional effects of sampling and preanalytical handling (Wan et al., 2017; Heitzer et al., 2019; Cescon et al., 2020). This constraint is especially important for methylation assays, for which rare informative molecules may be diluted by a large background of non-tumor cfDNA.

DNA methylation adds a complementary information layer because cancer-associated changes recur across loci and can retain cell- or tissue-of-origin information. Plasma methylation mapping supported tissue-origin deconvolution in the evaluated samples (Sun et al., 2015), while studies using targeted sequencing and cell-free methylated DNA immunoprecipitation sequencing (cfMeDIP-seq) reported cancer discrimination and classification from cfDNA methylation in the studied cohorts (Liu et al., 2018; Shen et al., 2018). Large targeted multi-cancer early detection (MCED), deep-methylation, and cell-type atlas studies extended these principles to broader cohorts and defined the normal-tissue background against which cancer-associated signals must be measured (Moss et al., 2018; Liu et al., 2020; Klein et al., 2021; Liang et al., 2021). Clinical evidence now spans colorectal screening, liver cancer surveillance, multi-cancer detection, and molecular residual disease (MRD), but performance remains indication- and stage-dependent.

These developments suggest a role for targeted sensing with clustered regularly interspaced short palindromic repeats (CRISPR) systems after biomarker discovery and validation. High-dimensional methylome sequencing, enrichment methods, fragmentomics, and machine-learning models can interrogate large feature spaces and are better suited to discovering and validating signatures. CRISPR is better suited to implementing compact, validated panels that can be read rapidly and reproducibly. Clinical methylation studies now provide independent validation, prospective intended-use evidence, and longitudinal benchmarks (Klein et al., 2021; Gao et al., 2023; Mo et al., 2023a; Wang et al., 2024; Nguyen et al., 2025; Patel et al., 2026; Zeng et al., 2026; Zhang et al., 2026). By contrast, most CRISPR-enabled methylation systems have been evaluated with synthetic targets, genomic DNA, cell lines, tissues, spiked matrices, or small exploratory patient cohorts.

The central technical problem is that CRISPR-associated (Cas) proteins are programmed mainly by sequence and structure, whereas 5-methylcytosine (5 mC) usually preserves Watson-Crick base pairing. Cas12a collateral cleavage provides signal gain but does not itself confer generic methylation sensitivity (Chen et al., 2018; Kaminski et al., 2021). Reported CRISPR-enabled approaches generate methylation discrimination primarily through sequence conversion (Li et al., 2019; Zhuang et al., 2024; Hu et al., 2026), methylation-sensitive or methylation-dependent nuclease gating (van Dongen et al., 2021; Wang et al., 2021; Wang X. et al., 2026), or native-state Cas modulation (Yang et al., 2025; Roth et al., 2026). Affinity-assisted recognition is considered here as an emerging design route because modified-DNA capture can in principle be coupled to downstream Cas confirmation, although direct 5 mC affinity-to-Cas evidence in cancer-associated cfDNA remains sparse. Engineered transduction can then recode an upstream state into an artificial protospacer-adjacent motif (PAM), single-stranded DNA (ssDNA) trigger, toehold, or device-level signal.

Similar endpoint signals can arise from architectures that preserve different fractions of the original cfDNA and place methylation specificity at different biochemical steps. An attomolar analytical limit of detection (LOD) after digestion and high-gain amplification is not equivalent to an amplification-free inhibition readout from native cfDNA. Neither metric predicts patient-level sensitivity without accounting for sample input, molecular recovery, methylation fraction, background, and decision thresholds. PAM-independent transduction can broaden targetability without making Cas intrinsically methylation-sensitive.

From a translational bioengineering perspective, the challenge extends beyond molecular discrimination. Scarce cfDNA molecules must survive sample preparation, methylation-state encoding, amplification or transduction, and device readout while retaining a quantitative relationship to the original biological state. Development therefore needs to connect biomarker selection and molecular recognition with signal transduction, device integration, and testing in clinically relevant matrices.

Existing reviews have addressed CRISPR diagnostics and locus-specific methylation sensing (Kaminski et al., 2021; Yu et al., 2023), cfDNA methylation (Luo et al., 2021), liquid-biopsy implementation (Ignatiadis et al., 2021; Valpione et al., 2026), fragmentomics (Lo et al., 2021; Bruhm et al., 2025), and cancer methylation biology (Koch et al., 2018). A recent review also surveys CRISPR technologies for DNA and RNA methylation across conversion-assisted, restriction-enzyme-mediated, and direct Cas-sensing strategies (Chen et al., 2026). However, these reviews do not fully address the path from a scarce, fragmented patient cfDNA molecule through methylation discrimination and signal transduction to the level of clinical evidence. The present review focuses on four questions. We first examine which methylation features are clinically informative and where methylated and unmethylated molecules become distinguishable. We then consider how that information is transferred to a Cas-readable output without unnecessary information loss and what specimen and clinical evidence supports each assay. We consider these elements separately from amplification, readout gain, device format, and evidence maturity so that molecular performance and device feasibility can be assessed independently.

Throughout this review, CRISPR-enabled methylation sensing denotes assays in which Cas contributes to detection or signal transduction. Conversion-free means that no base-conversion step is used. Native-state or direct Cas sensing is reserved for systems in which the preserved DNA modification alters the Cas-target interaction and provides methylation discrimination. We distinguish *in vitro* biochemical and cultured-cell experiments, animal models, human observational studies, and interventional clinical evaluations. Patient-derived material alone does not imply an interventional study or evidence of clinical benefit. Mechanistic evidence, analytical performance, specimen realism, clinical validation, clinical utility, and implementation are treated separately.

We use methylation fraction for the proportion of target molecules carrying a specified methylation state, with the locus or state across multiple cytosine-phosphate-guanine (CpG) sites and denominator stated explicitly. CpG density denotes the number of CpG sites within a defined sequence interval. Within-fragment methylation density denotes the number or proportion of those sites that are methylated. These quantities are distinct from the fraction of methylated molecules. The broader term methylation level is used only when a source reports an aggregate measure that cannot be assigned a more specific denominator. cfDNA denotes the entire cell-free DNA population, whereas ctDNA denotes its tumor-derived component.

## 2 Literature search and study selection

This mechanistic narrative review used a targeted, non-exhaustive literature-identification strategy. Searches were last updated on 31 August 2026. Literature identification drew on PubMed/MEDLINE and Web of Science Core Collection, supplemented by publisher websites, DOI verification, backward and forward citation tracking, and targeted engineering, microfluidics, materials, and biosensor searches. Search concepts combined CRISPR/Cas, DNA methylation (5 mC) or hydroxymethylation (5-hydroxymethylcytosine, 5hmC), cfDNA/ctDNA, cancer/liquid biopsy, and analytical or clinical validation terms. Query formulations were adapted to the specific question and to database syntax rather than applied as a single fixed Boolean protocol. Supplementary Table S1 records the databases, final search date, search concepts and recorded query formulations, coverage and eligibility rules, data-extraction fields, and evidence-classification criteria. These details document the targeted narrative-review strategy but do not imply PRISMA-style exhaustive screening or pooled evidence synthesis.

English-language peer-reviewed articles were eligible, with no lower publication-date limit. Preprints and grey literature were excluded. Articles available online with a permanent DOI by the coverage cutoff were eligible even when issue assignment occurred later. Primary studies were prioritized, especially those using patient-derived cfDNA. Reviews, guidelines, and non-CRISPR studies were retained for context, standards, or comparators. Studies of 5hmC were included only as mechanistic, interference, or affinity/transduction precedents, not as equivalent clinical evidence for cancer-associated 5 mC assays. Papers without an identifiable contribution to methylation discrimination, Cas-mediated detection, cfDNA biology, relevant biomarker validation, or analytical/clinical translation fell outside the review scope.

Searching PubMed/MEDLINE and Web of Science Core Collection, supplemented by publisher checks, DOI verification, and backward/forward citation tracking, broadened coverage and helped identify engineering studies with incomplete biomedical indexing. These measures do not remove selection bias. Choices of search terms, seed papers, and mechanistic relevance may favor well-cited or positive studies. This limitation precludes an exhaustive evidence claim. The A/B assignments are structured author appraisals under the criteria in Section 7.5, not validated evidence grades. Independent duplicate A/B rating was not performed, so inter-rater reliability was not estimated. Supplementary Table S2 records study characteristics and assignment rationales.

## 3 Biological constraints define the assay

### 3.1 Cancer-associated methylation patterns relevant to targeted detection

Cancer-associated methylation is attractive for targeted detection because differences between cancer and non-cancer samples recur at CpG-rich promoters, enhancers, developmental regulatory regions, and broader methylation domains (Koch et al., 2018). Such differences can identify candidate biomarkers without demonstrating that methylation caused the malignancy. A tumor-normal difference is only the starting point. For use in plasma, a biomarker must remain discriminative after dilution by normal cfDNA and across relevant benign or inflammatory backgrounds. Targeted loci such as SHOX2, SEPT9, SDC2, and BCAT1/IKZF1 illustrate the range of candidate markers (Kneip et al., 2011; Church et al., 2014; Pedersen et al., 2015; Young et al., 2021), whereas genome-scale studies identify many tissue- and cancer-associated differentially methylated regions.

For targeted CRISPR, the relevant feature may be a single CpG, a short methylation haplotype, or a compact set of informative loci. Selection should begin with clinical information content and background specificity rather than PAM, restriction-site, or guide convenience. Thresholds and marker combinations can materially change performance, but panel expansion needs measurable incremental value (Pedersen et al., 2015; Cai et al., 2021; Young et al., 2021; Pedersen et al., 2022; Park et al., 2026). A technology-first strategy can otherwise prioritize analytically convenient loci over clinically informative, recoverable targets.

### 3.2 cfDNA fragmentation, tumor fraction, and background methylation

Plasma cfDNA is fragmented and biologically structured. Nucleosome-footprinting and cancer fragmentomics studies show that fragment endpoints and size distributions carry tissue- and tumor-associated information (Snyder et al., 2016; Underhill et al., 2016; Mouliere et al., 2018; Cristiano et al., 2019; Lo et al., 2021; Bruhm et al., 2025). Separate analyses of methylation and fragmentation support links with chromatin accessibility and nuclease preference (An et al., 2023; Noë et al., 2024). An et al. (2023) combined human cfDNA observations with colorectal-cancer patient-derived xenograft mouse data. These evidence types inform mechanistic models but do not establish a universal causal pathway in human cancer. For direct targeting, the informative CpG, guide-binding interval, PAM, and any auxiliary-enzyme site must coexist on a recoverable fragment. Size selection can enrich the relative ctDNA fraction while discarding molecules (Underhill et al., 2016; Mouliere et al., 2018). At low copy number, absolute recovery matters as much as relative enrichment.

The non-tumor background is also structured. Methylation deconvolution studies show that most plasma cfDNA in healthy individuals derives from hematopoietic lineages, with additional contributions from endothelial and other tissues (Sun et al., 2015; Moss et al., 2018; Loyfer et al., 2023). Epigenetic specificity therefore requires two conditions. The assay must distinguish methylated from unmethylated molecules, and the selected methylation state must be sufficiently uncommon in relevant non-cancer tissues. Even perfect molecular discrimination cannot compensate for a biomarker that is frequently methylated in normal leukocytes or inflamed tissue.

Molecule availability often sets the sensitivity ceiling. Analysis of thousands of clinical plasma specimens shows that the probability of sampling enough target genome equivalents falls rapidly as target fraction declines and as more positive molecules are required for a confident call (Gamisch, 2026). Large-scale cfDNA sequencing also reveals locus-specific fragmentation-associated coverage biases that can reduce recoverability of some genomic regions (Vijayan Selvarajan et al., 2026). When the expected informative input approaches one molecule, Poisson sampling and upstream recovery dominate over reporter brightness.

These constraints create a finite molecular budget. A targeted assay is informative only when enough target molecules survive sampling, extraction, locus-specific fragmentation, and architecture-specific processing. We introduce this concept qualitatively here and formalize it with a simple Poisson bookkeeping model in the section “Molecule availability and stochastic sampling.”

### 3.3 Single-CpG versus regional and haplotype methylation information

Single-CpG assays simplify guide design, but tumor methylation often occurs in correlated short regions. Methylation haplotype blocks can match the scale of cfDNA fragments, and multi-CpG co-methylation has improved tissue deconvolution or cancer classification relative to average methylation or isolated CpGs in the reported datasets (Guo et al., 2017; Mo et al., 2023a). Read-level measures of epigenetic instability and integrated methylome-fragmentome analyses likewise indicate that within-fragment patterns can carry information beyond mean methylation (Thursby et al., 2026; Zeng et al., 2026). A short co-methylation pattern may therefore be rarer in normal tissue than any constituent CpG.

Regional information also carries a geometric cost because every required site increases the chance that a short plasma fragment is incomplete. The added biological specificity of multi-CpG information must be weighed against fragment recovery, guide competition, channel count, and additional molecular steps.

### 3.4 Clinically evaluated and emerging methylation biomarkers

SEPT9 illustrates why analytical detectability and screening performance must be separated. In prospective asymptomatic colorectal cancer (CRC) evaluation, plasma methylated SEPT9 showed substantially lower sensitivity, especially for stage I disease and advanced adenomas, than retrospective case-control studies suggested (Church et al., 2014). The assay itself was analytically validated at low methylated-DNA input (Potter et al., 2014). Differences in population, disease spectrum, assay configuration and evaluation design limit causal attribution of that discrepancy. This gap provides a useful benchmark when interpreting early CRISPR reports.

Targeted markers illustrate several intended uses. Examples include SHOX2 in lung cancer, SDC2 and BCAT1/IKZF1 in colorectal cancer, FAR1 in hepatocellular carcinoma (HCC), and compact multilocus tests for screening or recurrence (Kneip et al., 2011; Oh et al., 2013; Pedersen et al., 2015; Symonds et al., 2016; Murray et al., 2017; Cai et al., 2021; Jin et al., 2021; Young et al., 2021; Park et al., 2026). The relevant lesson for CRISPR is not that any single gene is optimal, but that biomarker performance depends on population, threshold, and clinical question. In the >100,000-participant ColonAiQ community study, incomplete colonoscopic verification of test-negative participants meant that positive predictive value and follow-up adherence were interpretable whereas full sensitivity and specificity were not (Wang et al., 2024). Selected clinical cfDNA methylation benchmarks are summarized in Supplementary Table S3.

High-dimensional classifiers are discovery resources rather than ready-made CRISPR panels. MCED classifiers, methylome-fragmentome resources, read-level metrics, and gastrointestinal models combine large feature sets (Liu et al., 2020; Klein et al., 2021; Main et al., 2026; Stutheit-Zhao et al., 2026; Thursby et al., 2026; Zeng et al., 2026; Zhu et al., 2026). Their discrimination may depend on distributed weak signals, correlations, tissue-of-origin information, and fragment-level patterns. Selecting a few strongly differential loci does not demonstrate that the smaller panel preserves the original classifier’s performance. Compact deployment therefore requires a new feature-selection and validation exercise, as discussed in Section 6.4.

### 3.5 Assay requirements imposed by liquid-biopsy biology

A clinically oriented assay therefore needs to preserve scarce fragmented molecules, reject structured non-tumor methylation background, and be validated in matrix- and fragment-relevant materials. Collection, processing, storage, extraction, and quality assessment can alter cfDNA yield and fragmentation (Kerachian et al., 2021; Harlé et al., 2026). Reference-material and validation studies further show that methylation fraction, recovery, limits of blank, detection and quantification, precision, and interference should be measured across the complete workflow (Yu et al., 2015; Deveson et al., 2021; Alexander et al., 2023; Lockwood et al., 2023). Fragment-matched, low-copy controls are more informative than fully methylated oligonucleotides alone. Representative conventional and non-CRISPR methylation methods that provide translational benchmarks for CRISPR-enabled assays are summarized in Table 1. Later sections develop the corresponding engineering and validation requirements.

**TABLE 1:** Representative DNA methylation methods as translational benchmarks for CRISPR-enabled biosensors.

Approach	Encoding and readout	Molecule recovery	Resolution and quantification	Relevance to CRISPR
Bisulfite qMSP or methylation-specific dPCR	Unmodified C becomes U/T; 5mC and 5hmC remain read as C. Standard bisulfite does not distinguish 5 mC from 5hmC	Conversion can fragment DNA and reduce recovery at low cfDNA input	Targeted loci/panels; dPCR supports absolute counting, while qMSP requires calibration	Clinical targeted benchmarks include SEPT9, SHOX2, SDC2 and BCAT1/IKZF1; calibration and intended use matter (Kneip et al., 2011; Oh et al., 2013; Church et al., 2014; Pedersen et al., 2022)
Targeted or genome-scale methylation sequencing	Conversion followed by targeted/broad sequencing and classification	Extraction, conversion and library preparation can lose molecules; longer fragments do not guarantee greater recovery (Kresse et al., 2025)	Base, regional and haplotype information; large multiplexed feature sets	Discovery and validation of MCED, tissue-of-origin and MRD signatures (Liu et al., 2018; Klein et al., 2021; Zeng et al., 2026)
Affinity enrichment with NGS or dPCR	Antibodies or MBD proteins enrich modified fragments without conversion	Native sequence retained; recovery depends on binder, CpG context and fragment length	Regional enrichment; absolute fraction requires calibrated input/recovery measurements	Non-Cas affinity precedent with plasma feasibility and enrichment bias (Nair et al., 2011; Shen et al., 2018; Truong et al., 2025)
Restriction-enzyme assays	Methylation state changes fragment integrity, termini or amplifiability	Avoids base conversion; requires compatible enzyme sites and complete digestion	Single-site or short-region analysis; calibrated digestion/counting supports quantification	Mechanistic benchmark for gating, with fragment-dependent digestion constraints (Zhen et al., 2025)
Native nanopore calling	Modification-sensitive ionic currents read on native DNA.	Retains sequence/modification; requires sequenceable molecules and library preparation	Single-base/haplotype calls depend on calibration, context and coverage	Comparator for direct native readout (Simpson et al., 2017; Yuen et al., 2021); CRISPR may offer a targeted implementation

Abbreviations: 5hmC, 5-hydroxymethylcytosine; 5mC, 5-methylcytosine; cfDNA, cell-free DNA; dPCR, digital polymerase chain reaction; LOD, limit of detection; MBD, methyl-CpG-binding domain; MCED, multi-cancer early detection; MRD, molecular residual disease; MSRE, methylation-sensitive restriction endonuclease; NGS, next-generation sequencing; qMSP, quantitative methylation-specific polymerase chain reaction.

## 4 From epigenetic state to CRISPR signal: a two-layer recognition and transduction framework

### 4.1 A two-layer information-flow model

We classify CRISPR-enabled methylation assays according to the first step at which methylated and unmethylated molecules become distinguishable. Sequence conversion recodes the modification as a sequence difference. Enzymatic gating converts methylation state into fragment integrity or reactive ends. Affinity-assisted recognition enriches or gates modified DNA, whereas native-state Cas modulation uses the preserved modification to alter Cas-target recognition. Direct affinity-assisted 5 mC-to-Cas evidence in cancer cfDNA remains sparse. Engineered molecular transduction is treated separately because it acts downstream of discrimination, for example by creating an artificial PAM, toehold, ssDNA trigger, or DNA-circuit state. The two-layer framework separates methylation specificity from signal gain and readout format (Figure 1).

**FIGURE 1:**
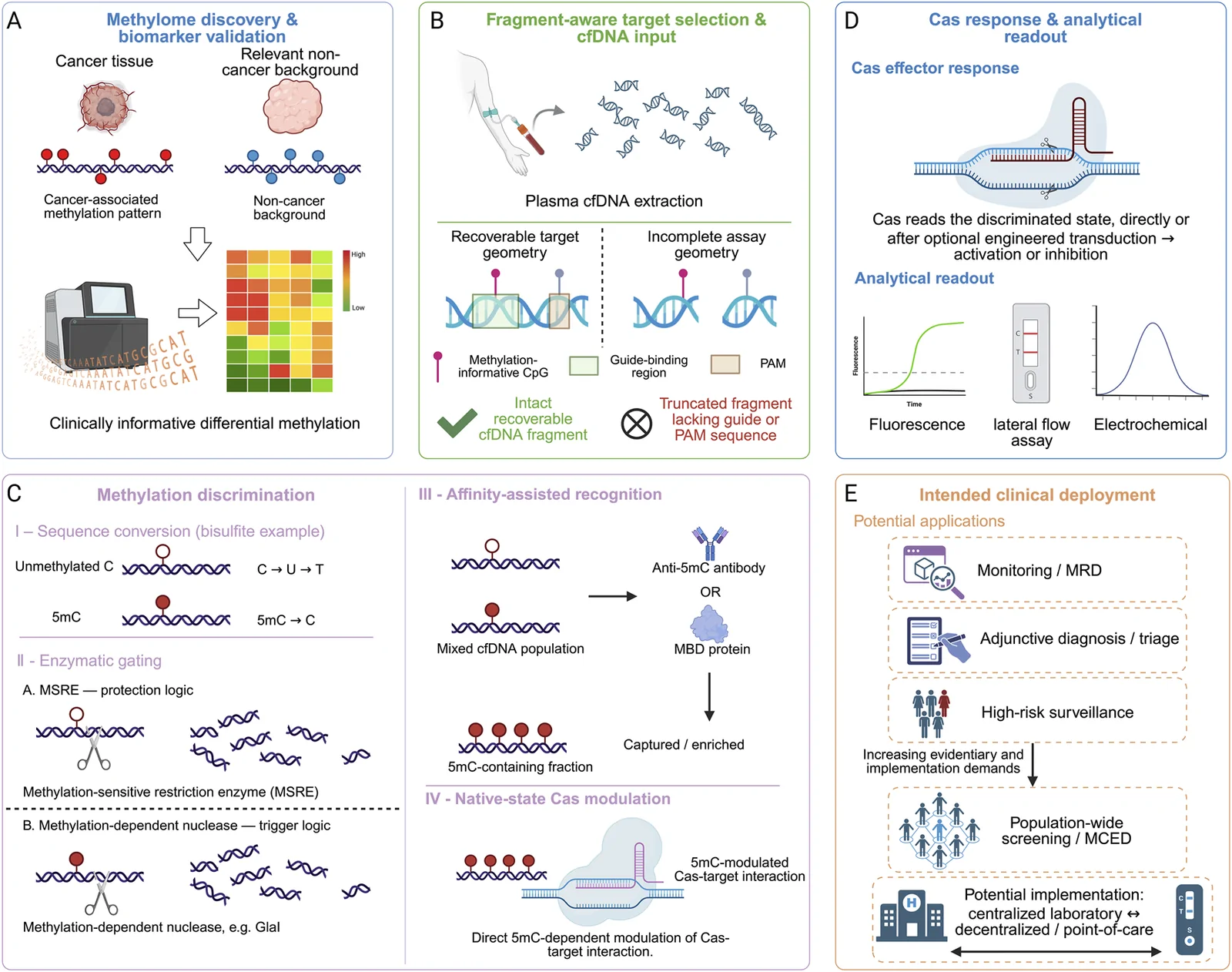
Biomarker-first framework for CRISPR-enabled biosensing of cancer-associated cfDNA methylation. **(A)** Discovery and validation of clinically informative methylation biomarkers against relevant non-cancer backgrounds. **(B)** Selection of fragment-recoverable cfDNA targets with compatible assay geometry. **(C)** Four primary routes for methylation discrimination: sequence conversion, enzymatic gating, affinity-assisted recognition, and native-state Cas modulation. Optional engineered transduction can further convert a discriminated methylation state into a Cas-readable input. **(D)** Differential Cas activation or inhibition is translated into analytical outputs. **(E)** Potential clinical deployment spans monitoring/MRD, adjunctive diagnosis or triage, high-risk surveillance, and, with increasing evidentiary and implementation demands, population-wide screening/MCED. Abbreviations: 5mC, 5-methylcytosine; Cas, CRISPR-associated protein; cfDNA, cell-free DNA; CRISPR, clustered regularly interspaced short palindromic repeats; DNA, deoxyribonucleic acid; MBD, methyl-CpG-binding domain; MCED, multi-cancer early detection; MRD, molecular residual disease; MSRE, methylation-sensitive restriction endonuclease; PAM, protospacer-adjacent motif.

### 4.2 Route I: sequence conversion

Sequence conversion provides a direct bridge from epigenetic state to sequence-programmed CRISPR. Bisulfite converts unmodified cytosine to uracil while 5mC and 5hmC remain largely read as cytosine, creating a sequence difference without distinguishing 5 mC from 5hmC (Nestor et al., 2010). HOLMESv2 paired this logic with Cas12b and quantitative readout in cell-line DNA (Li et al., 2019). Other platforms add padlock-based cascade amplification (Zhang L. et al., 2022; Figure 2B) or transcriptional transduction (Han et al., 2023), while meHOLMES uses TET2/APOBEC conversion before polymerase chain reaction (PCR)/Cas12a and engineered PAM logic (Zhuang et al., 2024). PAM-free and multiplex readouts further broaden targetability (Li et al., 2024; Hu et al., 2026).

**FIGURE 2:**
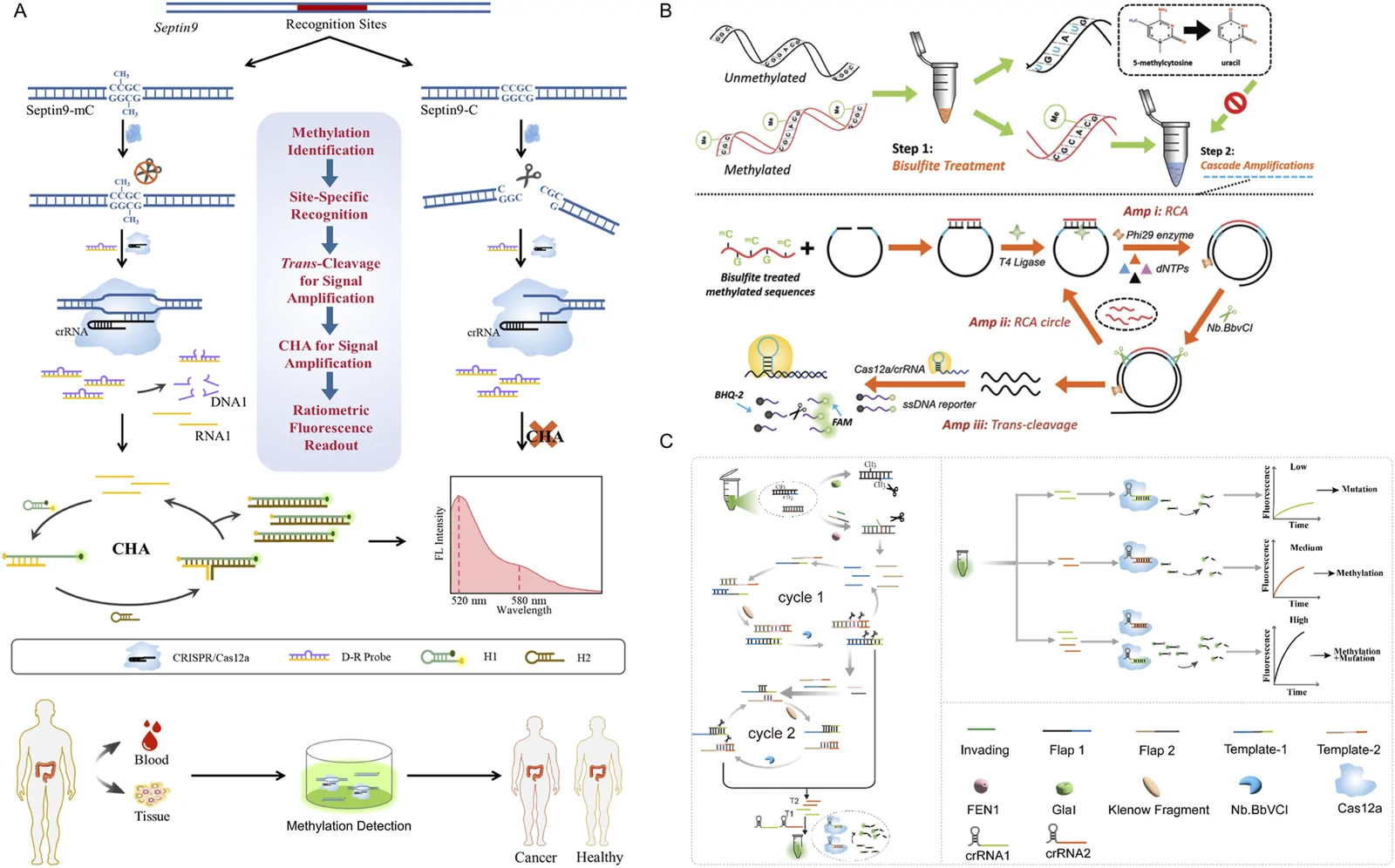
Representative CRISPR-enabled methylation sensing strategies spanning enzymatic gating, sequence conversion, and engineered signal encoding. **(A)** AciI-gated Cas12a sensing coupled to catalytic hairpin assembly (CHA), in which Cas12a trans-cleavage generates an RNA trigger for downstream CHA and ratiometric fluorescence readout of SEPT9 methylation (Ding et al., 2024). Copyright 2024 American Chemical Society. Adapted with permission. **(B)** The CRISPR-Cas12a-assisted methylation (CAM) assay, combining bisulfite conversion with padlock-mediated rolling-circle amplification, nicking-assisted recycling, and Cas12a trans-cleavage for cascade signal amplification (Zhang L. et al., 2022). Copyright 2022 The Royal Society of Chemistry. Adapted with permission. **(C)** A controllable Cas12a signal-logic-gate architecture for simultaneous analysis of SEPT9 methylation and KRAS G12C mutation, in which orthogonal enzymatic cascades generate distinct Cas12a activators and crRNA titration encodes the targets as graded fluorescence kinetic outputs (Wen et al., 2026). Copyright 2026 The Royal Society of Chemistry. Adapted with permission. Abbreviations and labels: CAM, CRISPR-Cas12a-assisted methylation assay; Cas, CRISPR-associated protein; CHA, catalytic hairpin assembly; CRISPR, clustered regularly interspaced short palindromic repeats; DNA/RNA, deoxyribonucleic/ribonucleic acid; crRNA, CRISPR RNA; ssDNA, single-stranded DNA; mC, methylcytosine; C, cytosine; CH3, methyl group; RCA, rolling-circle amplification; dNTPs, deoxynucleoside triphosphates; FAM, fluorescein; BHQ-2, Black Hole Quencher 2; FL, fluorescence; FEN1, flap endonuclease 1; FRET, Förster resonance energy transfer; H1/H2, hairpin probes 1/2; Amp, amplification; SEPT9/Septin9, septin 9; KRAS G12C, KRAS glycine-to-cysteine substitution at codon 12. DNA1/RNA1, D-R probe, flap, template and crRNA numerals identify source-specific oligonucleotides; Nb.BbvCI is a nicking endonuclease and Phi29 is a DNA polymerase.

Conventional bisulfite chemistry can degrade DNA and reduce library complexity, which is problematic for scarce, short cfDNA (Grunau et al., 2001). Bisulfite-free methods offer alternative routes to modified-base detection (Schutsky et al., 2018; Liu et al., 2019), but lower fragmentation does not necessarily imply higher molecule recovery. Kresse et al. (2025) compared specific enzymatic and bisulfite kits using healthy-donor cfDNA, fragmented colorectal-cancer cell-line DNA and colorectal-cancer plasma cfDNA. Enzymatic conversion preserved longer fragments but yielded less recovered DNA in that workflow. The apparent advantage therefore depends on the endpoint. The preferred method differed depending on whether the endpoint was fragment preservation or recoverable template yield for droplet digital PCR (ddPCR). These findings do not support a general ranking of all enzymatic and bisulfite workflows. Conversion chemistry, cleanup, input material and downstream readout require matched evaluation.

Conversion also reshapes CRISPR geometry. C-to-T recoding can create or destroy a PAM, alter guide complementarity, or change the targetable strand. Some methods exploit these changes, whereas others introduce artificial PAMs downstream (Zhuang et al., 2024; Hu et al., 2026). Amplification should occur only after the methylation state has been stably encoded, because copying native DNA before discrimination erases the modification information. For cfDNA, Route I is operationally demanding because performance depends on conversion efficiency, absolute recovery, fragment compatibility, and representation bias during post-conversion amplification.

### 4.3 Route II: enzymatic gating

Among the studies reviewed here, enzymatic gating has the broadest analytical evidence base. A methylation-sensitive restriction endonuclease (MSRE) or methylation-dependent nuclease first converts epigenetic state into fragment integrity, cleavage geometry, or an amplifiable end. Cas then recognizes the surviving or newly generated sequence and, where applicable, provides catalytic signal gain. Digestion avoids base conversion and can preserve the methylated base in the retained target, even when Cas never interrogates that base directly. Relative processing time and molecule recovery depend on the enzymes and protocols compared.

Restriction-site position can determine whether a gated molecule still activates Cas12a. Cuts near the PAM or seed are often disruptive, whereas more distal cuts may be tolerated (van Dongen et al., 2021). The dual methylation-sensitive restriction endonuclease/RPA-assisted CRISPR/Cas13a system (DESCS) used BstUI/HhaI gating before recombinase polymerase amplification (RPA), T7 transcription, and Cas13a readout and reported an 86.4 a.m. LOD and 0.01% methylation-fraction discrimination in controlled mixtures (Wang et al., 2021; Figure 3A). GlaI-based designs invert the logic by selectively cleaving methylated DNA to generate amplification triggers (Zhou et al., 2022b; Wu et al., 2023; Xie et al., 2026). Thus enzyme identity, CpG, PAM, guide, and fragment endpoints form one geometric design problem.

**FIGURE 3:**
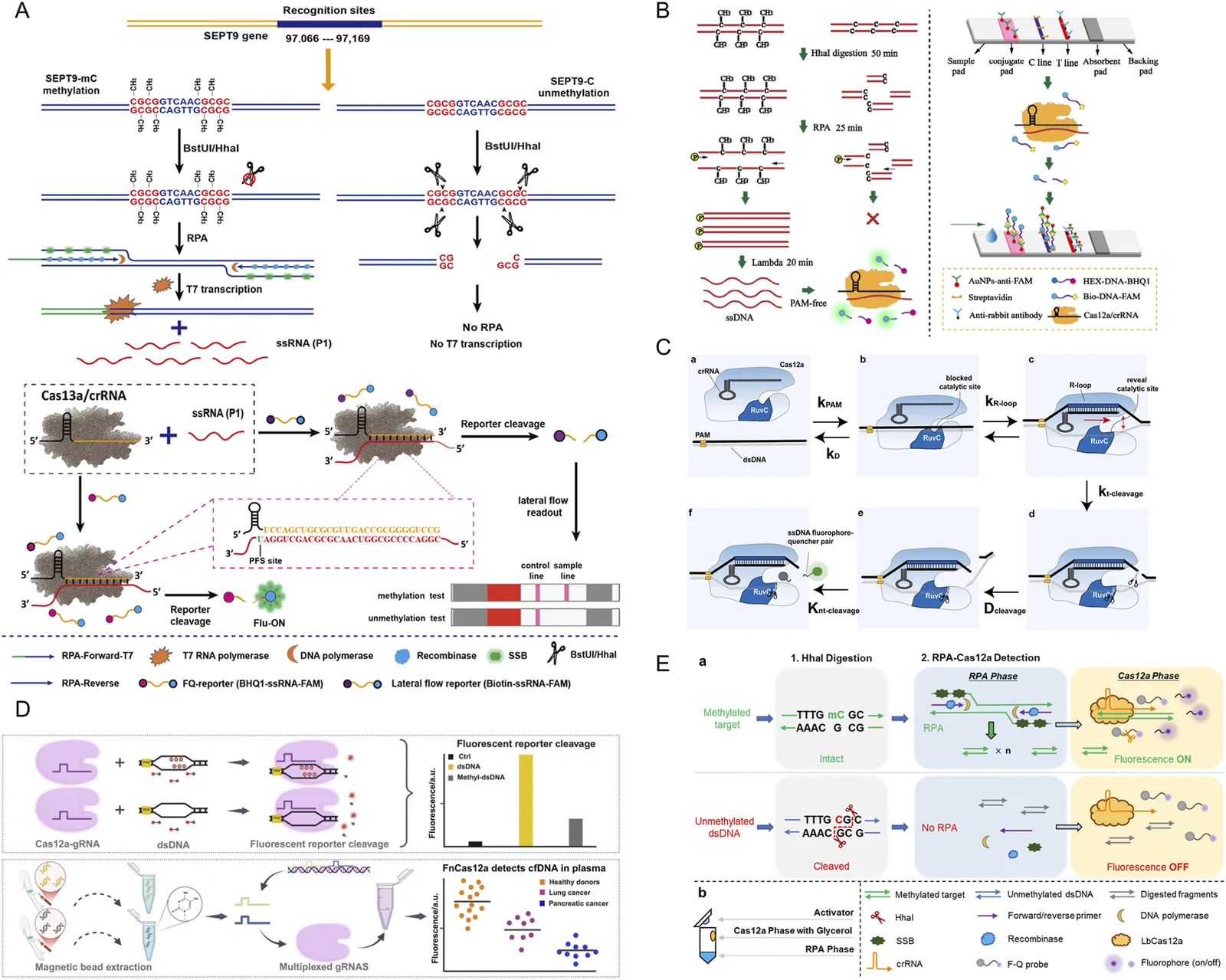
Representative CRISPR-enabled DNA methylation sensing architectures and mechanistic principles. **(A)** The dual methylation-sensitive restriction endonuclease/RPA-assisted CRISPR/Cas13a system (DESCS) uses dual BstUI/HhaI methylation-sensitive restriction digestion to preserve methylated SEPT9 DNA, followed by RPA, T7 transcription, and Cas13a collateral cleavage for fluorescence or lateral-flow readout (Wang et al., 2021). Copyright 2021 American Chemical Society. Adapted with permission. **(B)** The endonuclease-assisted PAM-free recombinase polymerase amplification/CRISPR/Cas12a assay (E-PfRPA/Cas) couples HhaI gating with RPA and Lambda exonuclease treatment to generate a PAM-independent ssDNA activator for Cas12a, enabling fluorescence and lateral-flow detection (Zhou et al., 2022a). Copyright 2022 American Chemical Society. Adapted with permission. **(C)** Canonical Cas12a target interrogation, illustrating PAM recognition, R-loop formation, target cleavage, and activation of collateral ssDNA cleavage (van Dongen et al., 2021). © 2021 The Authors. Published by Elsevier B.V. Adapted with permission. **(D)** CRISPR-MeDNA exploits site-specific 5 mC-dependent suppression of Cas12a trans-cleavage for amplification-free analysis of plasma cfDNA, including multiplexed-gRNA detection in cancer and healthy-donor samples (Yang et al., 2025). © The Author(s) 2025. Published by Oxford University Press. Adapted under the Creative Commons Attribution 4.0 International License (CC BY 4.0). **(E)** HhaI digestion and glycerol-enhanced RPA-CRISPR/Cas12a detection (HGRC) integrates HhaI gating with glycerol-enhanced, phase-separated RPA-Cas12a detection to improve amplification efficiency and fluorescence readout (Lu et al., 2024). © 2024 by the authors. Licensee MDPI, Basel, Switzerland. Adapted under the Creative Commons Attribution 4.0 International License (CC BY 4.0). Abbreviations and labels: 5mC, 5-methylcytosine; Cas, CRISPR-associated protein; cfDNA, cell-free DNA; CRISPR, clustered regularly interspaced short palindromic repeats; gRNA, guide RNA; PAM, protospacer-adjacent motif.

Enzymatic gating is often followed by multistep amplification or device-level gain. The endonuclease-assisted PAM-free recombinase polymerase amplification/CRISPR/Cas12a assay (E-PfRPA/Cas) uses HhaI discrimination, RPA, exonuclease generation of a PAM-free ssDNA activator, and Cas12a (Zhou et al., 2022a; Figure 3B). Other amplification modules include GlaI-based strand displacement (Xie et al., 2026), CRISPR-triggered catalytic hairpin assembly with ratiometric readout (Ding et al., 2024; Figure 2A), and cascade exponential amplification (Pataer et al., 2024; Figure 4A). Device- and reporter-level extensions include DNA/silver nanocluster fluorescence (Ling et al., 2026), nanozyme-iontronic gain (Fan et al., 2026; Figure 5A), glycerol-mediated phase separation for RPA-Cas12a compatibility (Lu et al., 2024; Figure 3E), organic electrochemical transistors (Xu et al., 2027), and semi-digital droplet readout (Huang et al., 2026). A nanoporous-gold HpaII-RPA-Cas12a electrochemical assay reported target-dependent LODs of 3.4–5.2 fM and 1% methylation-fraction discrimination in artificial plasma and blinded contrived specimens, without patient-derived cfDNA validation (Wang X. et al., 2026). A dual-HhaI/BstUI asymmetric-RPA design reported a 0.5 fM concentration LOD and 1% methylation-fraction discrimination in defined materials (Li et al., 2026b). These values characterize the complete gating-amplification/readout architecture rather than intrinsic Cas methylation selectivity.

**FIGURE 4:**
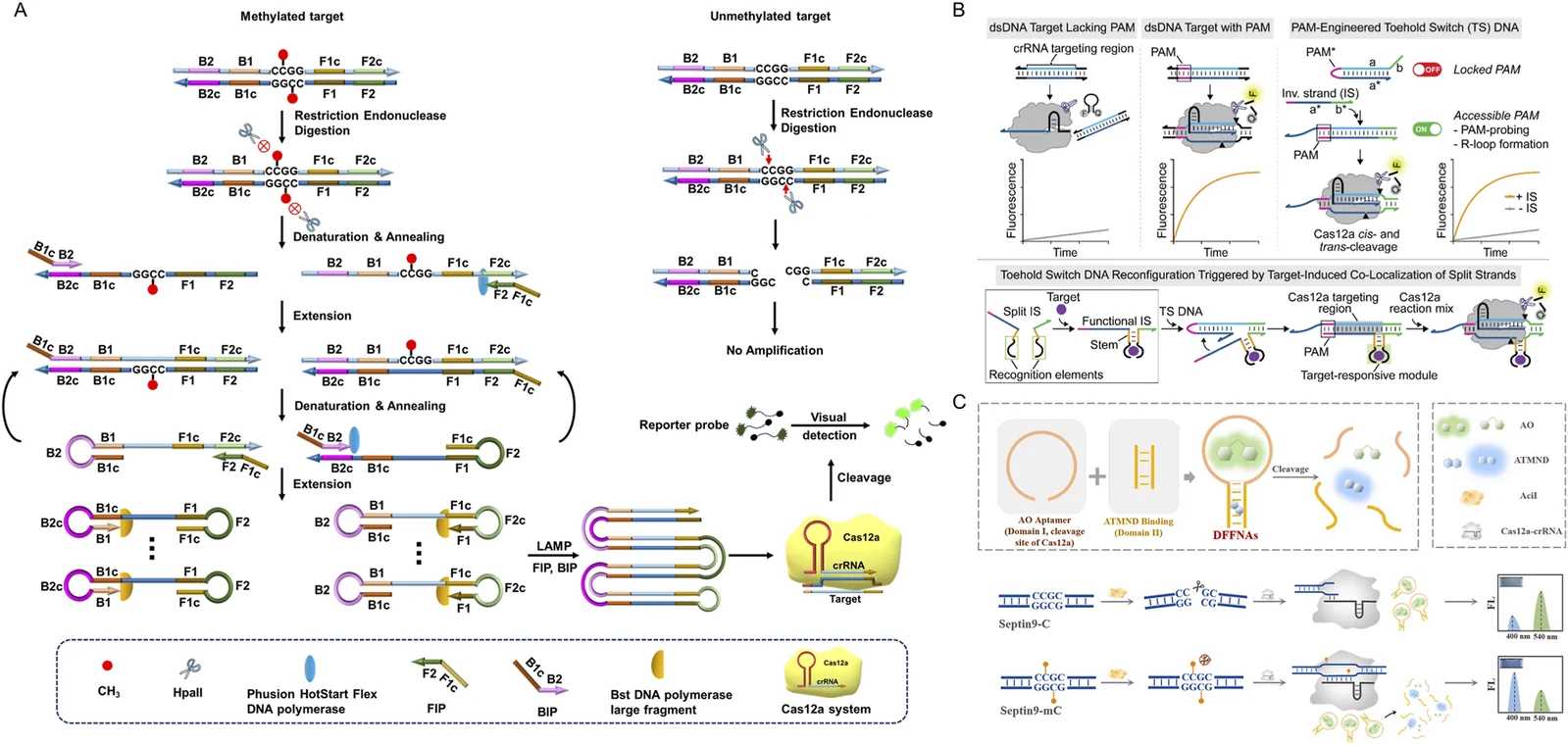
Representative amplification, engineered transduction, and readout modules relevant to CRISPR-enabled DNA methylation sensing. **(A)** Single-MSRE cascade exponential amplification, in which HpaII digestion suppresses amplification of unmethylated DNA, whereas intact methylated targets undergo cascade amplification followed by Cas12a-based visual readout (Pataer et al., 2024). Copyright 2024 American Chemical Society. Adapted with permission. **(B)** PAM-engineered toehold-switch DNA as an example of engineered molecular transduction: target-induced strand displacement reconfigures a locked hairpin, restores PAM accessibility, and activates Cas12a cis- and trans-cleavage (Bagheri et al., 2024). Copyright 2024 Wiley-VCH GmbH. Adapted with permission. **(C)** A label-free dual-fluorescence functional nucleic acid reporter coupled to AciI-gated Cas12a sensing of SEPT9 methylation, generating a ratiometric fluorescence output after Cas12a trans-cleavage (Tian et al., 2026). © 2025 The Authors. Published by Elsevier B.V. on behalf of KeAi Communications Co. Ltd. Adapted with permission. Abbreviations and labels: Cas, CRISPR-associated protein; CRISPR, clustered regularly interspaced short palindromic repeats; DNA, deoxyribonucleic acid; FIP/BIP, forward/backward inner primer; TS, toehold switch; IS, invading strand; AO, auramine O; ATMND, 5,6,7-trimethyl-1,8-naphthyridin-2-amine; DFFNAs, dual-fluorescence functional nucleic acids; FL, fluorescence; mC, methylcytosine; C, cytosine; CH3, methyl group; MSRE, methylation-sensitive restriction endonuclease; PAM, protospacer-adjacent motif; SEPT9/Septin9, septin 9. F1/F2/B1/B2 and their c-suffixed labels identify primer-binding regions and complementary regions; a, a*, and b denote strand domains. F and Q indicate fluorophore and quencher. Bst denotes the DNA polymerase used in the source amplification scheme.

**FIGURE 5:**
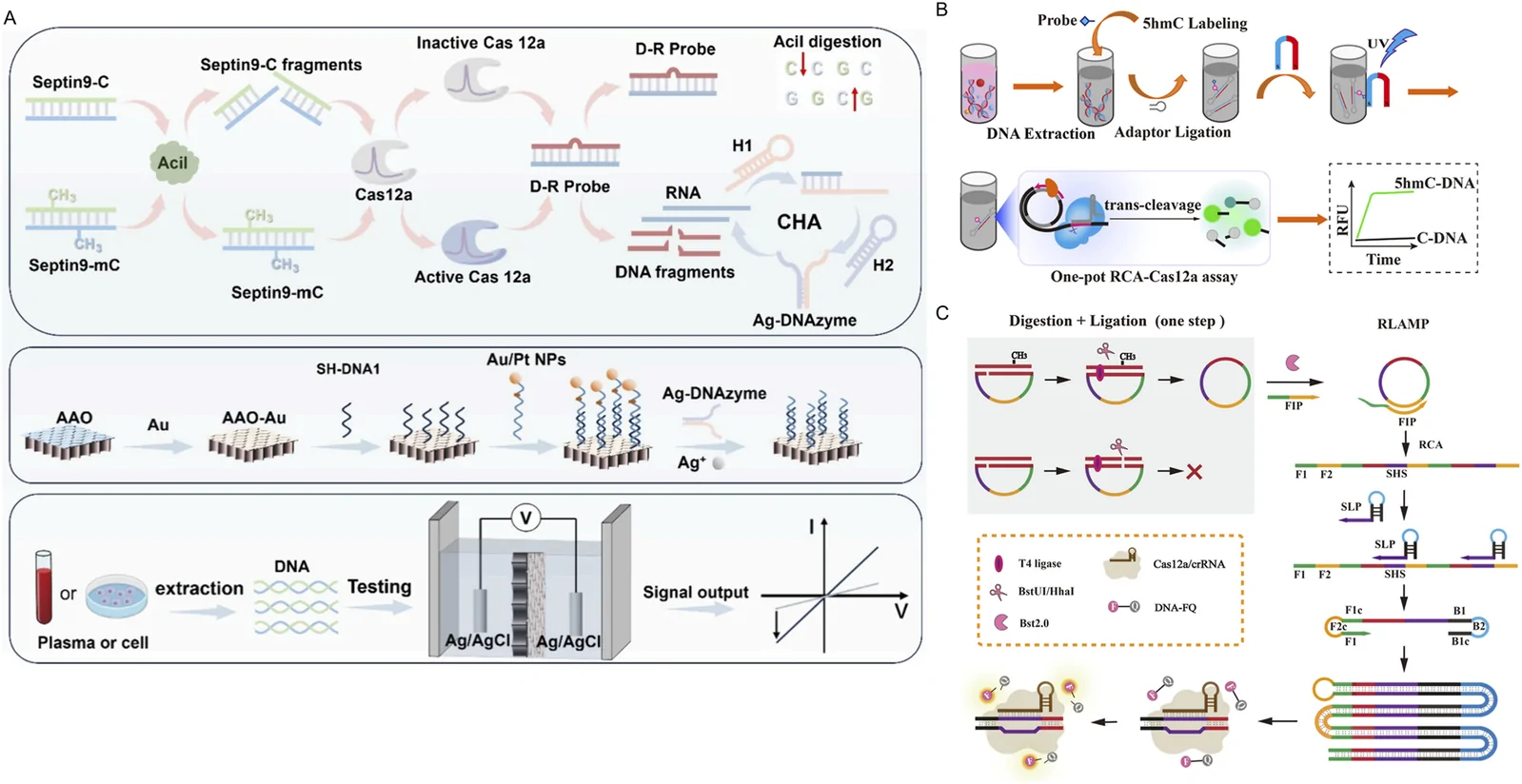
Representative advanced CRISPR-enabled architectures for methylation and hydroxymethylation sensing. **(A)** AciI-gated Cas12a sensing coupled to a CHA-Ag-DNAzyme cascade and Au/Pt nanozyme-regulated iontronic readout for SEPT9 methylation detection (Fan et al., 2026). Copyright 2026 American Chemical Society. Adapted with permission. **(B)** Chemically assisted one-pot RCA-Cas12a (CAICas12a) detection of 5-hydroxymethylcytosine (5hmC), combining selective chemical labeling and enrichment with adaptor ligation, rolling-circle amplification, and Cas12a fluorescence readout (Zou et al., 2025). Copyright 2025 American Chemical Society. Adapted with permission. **(C)** DL-RLAMP/Cas12a sensing of p15 methylation. HhaI/BstUI digestion and padlock ligation are combined in one step, followed by rolling circle extension-assisted loop-mediated isothermal amplification and a separate Cas12a readout. Adapted from Scheme 1 in the original publication by Zhou et al. (2026). Copyright 2026 The Royal Society of Chemistry. Adapted with permission. Abbreviations and labels: 5hmC, 5-hydroxymethylcytosine; AAO, anodic aluminum oxide; Cas, CRISPR-associated protein; CHA, catalytic hairpin assembly; CRISPR, clustered regularly interspaced short palindromic repeats; DNA, deoxyribonucleic acid; Au, gold; Pt, platinum; Ag, silver; NPs, nanoparticles; SH-DNA1, thiolated DNA probe 1; H1/H2, hairpin probes 1/2; RCA, rolling-circle amplification; DL, digestion-ligation; RLAMP, rolling circle extension-assisted loop-mediated isothermal amplification; SLP, stem-loop primer. SHS denotes the engineered sequence domain that yields the Cas12a-recognized amplicon; SHSc is its complementary domain in the padlock template. F1/F2/B1/B2 denote primer-binding sequence domains, and the suffix c denotes a complementary domain. DNA-FQ is a fluorophore-quencher-labeled DNA reporter. Bst2.0 denotes Bst 2.0 DNA polymerase; T4 ligase denotes T4 DNA ligase. D-R probe is the source-specific probe label in **(A)**. Ag/AgCl denotes the silver/silver-chloride electrode. Panel B is a 5hmC transduction precedent, not evidence for direct 5 mC affinity-to-Cas detection in patient cfDNA.

Specimen evidence remains limited and heterogeneous. Published studies include cervical exfoliated-cell DNA (Xu et al., 2023), cell-line, tissue, or peripheral-blood genomic DNA (Lu et al., 2024; Ding et al., 2025; Tian et al., 2025; Xie et al., 2026), serum-like matrices (Xu et al., 2027), and small patient-plasma cohorts (Fan et al., 2026; Hou et al., 2026). Patient-derived cfDNA evidence is therefore still exploratory, and none of the Route II studies identified here has reached independent locked-assay validation. The relevant translational question is whether digestion, recovery, and calibration remain reproducible in fragmented patient cfDNA, not simply how low a nominal LOD can be reported.

Incomplete digestion is a central failure mode, but its consequence depends on assay logic. In protection-based designs, an unmethylated molecule that escapes a nuclease intended to remove it can become a false methylation-positive after RPA, loop-mediated isothermal amplification (LAMP), strand-displacement amplification (SDA), or another high-gain amplification step. In methylation-dependent trigger designs, incomplete cleavage of methylated molecules can reduce trigger generation and cause false-negative or underestimated signals. Dual enzymes, protected-primer designs, padlock logic, and sequential gates can reduce leakage or missed cleavage, but they add components, reaction time, and lot-to-lot variability. The breadth of Route II reports does not demonstrate superior performance under matched conditions. Reproducible digestion and molecule recovery remain priorities alongside analytical sensitivity.

### 4.4 Route III: affinity-assisted recognition

Affinity-assisted recognition uses methyl-CpG-binding domain (MBD) proteins, anti-5mC antibodies, or related binders to capture or gate modified DNA without base conversion or target cleavage. MBD-based gating (Ishidoya et al., 2024), immunoprecipitation coupled with digital PCR (dPCR) (Truong et al., 2025), and MBD-functionalized enrichment (Li et al., 2026a) provide precedents for recognition or low-input handling with non-Cas readouts. A chemical-labeling/affinity Cas12a assay for 5hmC provides a modified-base-to-Cas precedent (Zou et al., 2025; Figure 5B). These studies answer different questions. Neither non-CRISPR 5 mC enrichment nor 5hmC-to-Cas detection demonstrates an end-to-end 5 mC affinity-to-Cas assay in patient cancer cfDNA. Direct evidence for that combination remains limited among the studies identified here.

A recent non-CRISPR example used MBD-enriched plasma cfDNA sequencing to derive a 12-region CRC model (Qu et al., 2026). Its retrospective, single-center held-out comparison included only four CRC cases and five healthy controls (area under the curve 0.85; 95% confidence interval 0.579–1.000). Elevated scores in polyp samples leave disease specificity uncertain. This supports further evaluation of affinity-derived markers, not clinical validation of affinity-to-Cas detection.

Capture is not uniform across methylated molecules. In a direct comparison, methyl-DNA immunoprecipitation (MeDIP) and MBD capture preferentially recovered different CpG contexts, with MBD favoring higher CpG density (Nair et al., 2011). CpG density, within-fragment methylation pattern, and fragment length should therefore be treated as separate calibration variables. A longer fragment can present more binding sites, while a short fragment may lose a required binding context. The direction and magnitude of bias depend on the binder and assay conditions. An enrichment factor measured on one substrate should not be transferred to another.

Binding, washing, and elution also determine which captured molecules reach the Cas reaction. Strong binding does not guarantee efficient elution, and residual capture reagents may alter downstream activity. Enrichment changes the composition of the recovered pool, so the methylated fraction after capture need not equal the fraction in the starting cfDNA. Amplification of that pool preserves the selection bias and may add representation bias. A future affinity-to-Cas assay should measure input, flow-through, and eluate with fragment- and CpG-matched standards, assess matrix inhibition, and compare calibrated fractions with an orthogonal method. These are proposed validation requirements, not capabilities already demonstrated for patient 5 mC affinity-to-Cas sensing.

### 4.5 Engineered molecular transduction: an orthogonal enabling layer

Engineered molecular transduction takes an already discriminated methylation-dependent event and recodes it as a Cas-readable sequence or structure, such as an artificial PAM, toehold, ssDNA trigger, or DNA-circuit state. It is an enabling layer rather than a fifth source of methylation specificity. Its practical value lies in relaxing unfavorable biomarker geometry or changing signal direction and multiplexing options.

PAM-engineered toeholds and sticky-end double-stranded DNA (dsDNA) demonstrate Cas12a activation by engineered inputs in the tested configurations (Zhang W. et al., 2022; Bagheri et al., 2024; Figure 4B). Methylation workflows use the same principle when conversion or padlock amplification creates PAM-bearing products (Zhuang et al., 2024; Zhou et al., 2026; Figure 5C), or when nuclease gating generates PAM-free ssDNA activators (Zhou et al., 2022a). These approaches can adapt CRISPR to a validated biomarker without selecting a less informative locus solely because it has more convenient native geometry.

Transduction can also change how an upstream state is encoded at the readout. Examples include ratiometric fluorescence with dual-fluorescence functional nucleic-acid reporters (Tian et al., 2026; Figure 4C), mutation-methylation logic gates (Wen et al., 2026; Figure 2C), iontronic interfaces, DNAzyme cascades, and nanozyme-assisted colorimetry (Li et al., 2024; Tian et al., 2025; Fan et al., 2026; Wang Z. et al., 2026). Output modality alone does not identify the source of methylation specificity. Different readouts may share the same upstream discriminator, while similar readouts can implement different information logic.

Each transduction step introduces additional opportunities for molecule loss, signal leakage, and calibration error. At low target copy number, a large amplified signal can conceal substantial stochasticity in how many molecules successfully traversed the workflow. Transduction is therefore most useful when it expands targetability or information encoding without compromising the reproducible mapping between input molecules, methylation fraction, and output signal.

### 4.6 Route IV: intrinsic or native-state Cas modulation

Route IV uses the preserved modification itself as the primary discriminator. In specific Cas-target configurations, 5 mC changes recognition or activity without upstream base conversion or auxiliary methylation recognition (Yang et al., 2025; Roth et al., 2026). This remains a research direction supported by mechanistic evidence and exploratory observations in patient-derived cfDNA. It has not been established as a clinically validated alternative to conversion or enzymatic gating. Section 5 examines its context dependence, required controls, and current evidence limits.

### 4.7 Cross-route comparison: mechanistic directness versus evidence maturity

The reported evidence does not support a uniform ranking of routes. Sequence conversion and enzymatic gating have the broadest analytical evidence. Affinity-assisted 5 mC-to-Cas sensing remains sparse. Native-state sensing is mechanistically direct but context-dependent and clinically immature. Engineered transduction can expand targetability or readout options, but it does not provide an independent source of methylation specificity. Table 2 summarizes these trade-offs, Table 3 defines the evidence categories, and Supplementary Table S2 provides representative study-level characteristics and evidence assignments.

**TABLE 2:** Two-layer framework for CRISPR-enabled DNA methylation biosensing.

Framework element	Primary discriminator or transduction step	Role of Cas	Key trade-offs and evidence maturity
Primary route I: Sequence conversion	Bisulfite or enzymatic conversion recodes the modification as a sequence difference	Cas reads the converted sequence and provides sequence confirmation and/or catalytic signal amplification	Strong sequence programmability; liabilities include DNA/recovery loss, conversion efficiency, and amplification dependence. CRISPR implementations remain mostly analytical/biological proof-of-concept
Primary route II: Enzymatic gating	An MSRE or methylation-dependent nuclease changes fragment integrity or creates a methylation-dependent end	Cas recognizes the surviving or newly generated sequence and, where applicable, provides catalytic signal gain	Broadest CRISPR analytical evidence base among the primary routes reviewed here. Restricted by enzyme sites, incomplete digestion, fragment geometry, and leakage
Primary route III: Affinity-assisted recognition	MBD proteins, anti-5mC antibodies, or related binders enrich, capture, or sterically gate modified DNA.	Cas may provide downstream sequence confirmation, although many current precedents use non-Cas readers	Emerging route that preserves native sequence and relaxes restriction-site constraints, but capture efficiency and CpG/fragment-length bias can distort quantification. Direct affinity-to-Cas evidence for 5 mC in cancer-associated cfDNA remains sparse
Primary route IV: Native-state Cas modulation	In specific tested configurations, native 5 mC alters Cas recognition or cleavage; sensitivity is not uniform across targets or orthologs	Cas acts as both methylation discriminator and signal generator through differential activation or inhibition	Mechanistically direct and avoids upstream state conversion, but response is context- and ortholog-dependent; inhibition-based readouts require strong controls. Current patient-derived cfDNA evidence remains preliminary
Orthogonal enabling layer: Engineered molecular transduction	An already discriminated methylation-dependent event is recoded into an artificial PAM, toehold, ssDNA trigger, or DNA-circuit state	Cas responds to the engineered sequence or structure	Orthogonal enabling layer that expands targetability, signal direction, or multiplexing; additional components increase kinetic complexity, leakage, and calibration burden

**TABLE 3:** Proposed two-axis translational evidence framework for CRISPR-enabled methylation biosensors.

Evidence category	Defining material/design	Defensible claim
Axis A- A1. Synthetic target	Designed oligonucleotide or synthetic dsDNA with controlled methylation state	Mechanistic or analytical discrimination under defined conditions; not biological-matrix or clinical performance
Axis A- A2. Constructed/reference DNA	Plasmid, reference genomic material, experimentally methylated genomic DNA, or defined amplicon without reliance on an endogenous disease-associated state	Locus discrimination in a defined complex DNA background; not endogenous biological-state or patient-cfDNA performance
Axis A- A3. Endogenous biological DNA	Cell-line, primary-cell, exfoliated-cell, or tissue DNA carrying endogenous methylation states	Endogenous-state observations or discrimination in the tested cell/tissue model; not evidence of liquid-biopsy performance
Axis A- A4. Spiked biofluid/cfDNA surrogate	Synthetic, reference, or biological target added to plasma/serum or an extracted cfDNA surrogate	Matrix/extraction feasibility under the tested simulated conditions; not patient disease specificity
Axis A- A5. Patient-derived cfDNA	Clinical plasma/serum cfDNA tested without substituting constructed targets for patient molecules	Feasibility or association in patient-derived cfDNA; clinical validity depends on the independent clinical-validation axis
Axis B- B1. Exploratory patient-level association	Exploratory patient cohort, often small or single-center, with *post hoc* or incompletely locked decision rules	Exploratory patient-level association or proof-of-concept; not generalizable clinical validity
Axis B- B2. Independent locked-assay validation	Independent cohort evaluated against a comparator with reagent composition, panel, protocol, analysis, quality-control rules and threshold fixed before validation testing; no tuning to validation outcomes	Patient-level clinical validity in the studied setting; not evidence of clinical utility
Axis B- B3. Prospective intended-use validation	Prospectively enrolled intended-use population evaluated using a locked assay and prespecified decision rule, with the validation cohort independent of assay development where feasible	Performance in the intended population; does not by itself establish improved outcomes
Axis B- B4. Interventional clinical utility	Test-guided intervention compared with standard care or another management strategy	Whether use of the test improves clinical decision-making or patient outcomes
Axis B- B5. Real-world implementation	Routine deployment across laboratories, sites, or care pathways after validation	Whether the validated test can be implemented reproducibly and sustainably in routine care

Route selection is best viewed as a choice of where information loss and bias are introduced. Sequence conversion incurs its principal cost during chemical or enzymatic processing and molecule recovery. Enzymatic gating is constrained by restriction-site geometry and digestion completeness. Affinity-assisted recognition preserves native sequence but is affected by capture efficiency and CpG- or fragment-dependent enrichment bias. Native-state sensing avoids upstream state conversion but introduces context-dependent Cas responses and target-specific calibration. Engineered molecular transduction can broaden targetability or change signal logic, but it cannot restore methylation information lost before the transduction step. For low-copy cfDNA, the most suitable architecture is not necessarily the one with the largest endpoint signal. It is the architecture that preserves the greatest fraction of clinically informative molecules while maintaining adequate specificity and quantitative reproducibility.

Native-state readout is not unique to CRISPR. Nanopore sequencing can infer 5 mC from modification-sensitive ionic-current signals, with errors that depend on the caller, sequence context, and calibration (Simpson et al., 2017; Yuen et al., 2021). Nanopore therefore provides a useful comparator for mechanistic directness, whereas CRISPR may offer a more compact and programmable targeted implementation.

Mechanistic directness, analytical performance, and clinical evidence are separate dimensions. An indirect architecture may be translationally credible, whereas a direct one may remain exploratory. This distinction is important when interpreting reported LODs and claims of rapid, universal, or point-of-care methylation detection.

## 5 Emerging native-state recognition of 5 mC by Cas

### 5.1 Evidence that DNA modifications alter Cas recognition

Native-state methylation sensing is mechanistically plausible because Cas activation is not a single base-pairing event. Target interrogation includes PAM recognition, local DNA deformation, strand separation, R-loop propagation, conformational checkpoints, and catalytic activation (Figure 3C). Studies of Cas12a DNA clamping, DNA bending, and the bridge helix did not test methylation directly (Naqvi et al., 2022; Soczek et al., 2025; Ganguly et al., 2026). However, they identify structural checkpoints through which local modification-dependent DNA mechanics or protein-DNA contacts could plausibly alter cleavage or collateral activation. Cas12a collateral cleavage could then amplify a small modification-dependent difference generated during target interrogation (Chen et al., 2018).

The *in vitro* findings on native methylation sensitivity are not uniform. Van Dongen et al. (2021) reported no significant effect of methylation itself on average Cas12a trans-cleavage in their tested configuration. In that study, discrimination relied on restriction-enzyme gating. CRISPR-MeDNA subsequently reported site-dependent suppression of Cas12a activity (Yang et al., 2025). These differing results argue against a universal methylation response, although their causes cannot be assigned without matched comparisons of orthologs, guides, substrates and reaction conditions. ThermoCas9 adds evidence for methylation-sensitive PAM recognition in a different Cas system (Roth et al., 2026). Each proposed diagnostic configuration therefore requires its own validation.

### 5.2 Site, strand, density, and spacing dependence of 5 mC effects

In CRISPR-MeDNA *in vitro* experiments, 5 mC effects varied with target geometry. Cas12a inhibition depended on the position of the modified cytosine relative to the guide and PAM and on whether the modification resided on the target or non-target strand (Yang et al., 2025). The spacing of multiple methylated sites also influenced the response. Non-target-strand modifications produced stronger effects in several tested contexts, and combinations of CpGs could yield greater discrimination than a single modified site. The data are therefore more consistent with an analog, context-dependent response than with a universal methylated/unmethylated switch.

Context dependence has two design implications. Native sensing must be specified by locus, strand orientation, local sequence, Cas ortholog, guide register, and fragment boundaries. A biomarker coordinate alone is insufficient. Calibration is likewise target-specific. A 30% reduction in trans-cleavage at one CpG cannot be converted to methylation fraction using a curve established for another locus.

Density and spacing may offer an additional design opportunity. If multiple methylated cytosines on the same short cfDNA fragment collectively alter Cas activation, native sensing could in principle report a regional state without converting each CpG into a sequence difference. This possibility is relevant to methylation haplotypes, but it has not been established as a robust clinical regional readout and remains a testable design direction.

### 5.3 Cas ortholog dependence and mechanistic basis

Ortholog dependence is both a constraint and an opportunity for engineering. CRISPR-MeDNA reported different modification sensitivities among Cas12a orthologs, consistent with a role for protein-DNA contacts and conformational energetics in determining how strongly 5 mC is translated into altered trans-cleavage (Yang et al., 2025). ThermoCas9 biochemical and structural experiments support inhibition by methylation within specific PAM contexts, whereas methylation at the tested protospacer positions had no notable effect. Editing experiments in human cell lines support activity in those cellular models (Roth et al., 2026). They do not provide diagnostic evidence from patient-derived cfDNA or evidence of clinical treatment effects. The bridge-helix study of FnoCas12a and the DNA-bending work did not test methylation. They nevertheless identify conformational checkpoints that could plausibly respond to local, modification-dependent DNA mechanics (Soczek et al., 2025; Ganguly et al., 2026).

These mechanistic studies should not be treated as surrogate diagnostic validation. Their main value is hypothesis generation. Screening Cas orthologs or engineered variants for large differential responses between methylated and unmethylated targets may produce better epigenetic discriminators than simply selecting the most active wild-type nuclease. PAM-relaxed Cas12a variants already expand targeting ranges for genome and epigenetic editing, showing that protein engineering can alter accessible sequence space (Kleinstiver et al., 2019). A logical next step is to engineer a controlled methylation-response function as well as targetability.

### 5.4 CRISPR-MeDNA: demonstrated scope and current evidence

CRISPR-MeDNA provides exploratory evidence from patient-derived cfDNA for amplification-free native-state sensing (Yang et al., 2025; Figure 3D). Synthetic experiments showed site-, strand-, density-, and spacing-dependent Cas12a inhibition. At the CCDC140 promoter, the reported two-guide discrimination boundary was approximately 100 fM, compared with approximately 10 pM using a single guide. The defined-target workflow included synthetic DNA spiked into DNA-free plasma and subsequent extraction. The 1% methylated-fraction experiment used 1 nM total target DNA. That concentration and fraction result must not be interpreted as a 1% clinical ctDNA detection limit.

The patient experiment used cfDNA extracted from 4 mL of plasma and approximately 2 ng cfDNA in a 20 μL Cas12a reaction. The cohort comprised 15 healthy donors, 9 patients with lung cancer, and 10 with pancreatic cancer. Fluorescence after 30 min differed between groups (Yang et al., 2025). The study did not establish a prespecified clinical threshold, receiver-operating-characteristic performance, independent validation, or a benign/inflammatory control cohort.

The controlled *in vitro* experiments support modification-dependent Cas modulation. The observational patient comparison supports assay feasibility and shows a group-level difference in fluorescence within the sampled cohort. It does not isolate a cancer-specific causal explanation for the signal difference. It also does not establish clinically usable diagnostic sensitivity or specificity. The native-state route should therefore remain an exploratory research strategy until controlled analytical studies and independent, locked-assay patient validation resolve its quantitative and disease-specificity limitations.

### 5.5 Remaining evidence gaps

An inhibition-based native readout needs controls that distinguish methylation from loss of general assay activity. Matrix effects should be tested with matched buffer and extracted-plasma spike-ins, an extraction blank, and dilution or spike-recovery experiments. A noncompeting exogenous activation control in a separate aliquot can reveal general reaction suppression without consuming the scarce target in the analytical reaction. A low control signal should trigger a prespecified invalid-result rule rather than a methylation-positive call.

Nuclease or guide instability should be assessed with methylation-independent positive controls across storage conditions, reagent lots, and runs. A paired guide geometry shown experimentally to be insensitive to methylation can help normalize target abundance and general Cas activity. It is not automatically a valid denominator. Its locus recovery, guide accessibility, dynamic range, and response to matrix must also be matched. Orthogonal locus quantification can distinguish target scarcity or incomplete fragments from methylation-dependent inhibition. Ratio uncertainty becomes substantial when either signal approaches the blank.

Modification specificity should be challenged with otherwise matched unmethylated, 5 mC-containing, and 5hmC-containing fragments, including mixtures at relevant concentrations and positions. Other cytosine modifications should be tested when relevant to the target or preprocessing chemistry (Cui et al., 2020; Zou et al., 2025). Defined methylation-fraction gradients should be crossed with DNA input, fragment length, and plasma matrix. Healthy, benign, and inflammatory controls are then needed to distinguish modification sensitivity from cancer specificity. These experiments are recommendations for future validation. They were not all performed in the exploratory native-state studies.

### 5.6 Opportunities for Cas protein and guide engineering

A useful engineering objective is a reproducible differential response to methylated versus unmethylated DNA, not maximal Cas activity. Cas variants can be screened for this response, guides can place informative CpGs at modification-sensitive checkpoints, and paired methylation-sensitive and -insensitive geometries can control general enzyme suppression. Structural and PAM-relaxation studies identify tractable checkpoints for such co-design (Kleinstiver et al., 2019; Ganguly et al., 2026).

## 6 Biomarker-to-biosensor engineering under cfDNA constraints

### 6.1 Biomarker selection, tissue specificity, and fragment recoverability

The biological constraints above support a biomarker-first workflow. Candidate methylation features should first be selected based on validated clinical relevance and acceptable specificity against non-cancer backgrounds. The informative region should then be confirmed as recoverable in plasma cfDNA before CRISPR geometry is optimized. Convenient guide or PAM geometry cannot compensate for limited clinical specificity. Conversely, a clinically informative biomarker may be unsuitable for direct interrogation if the required region is rarely recovered intact. Longitudinal applications also require the selected feature to remain sufficiently stable during treatment and clonal evolution to support repeated measurement. Once a candidate region passes these biological filters, biosensor development can focus on guide register, strand orientation, PAM availability, auxiliary sites, and downstream transduction.

### 6.2 CRISPR accessibility: guide, PAM, and auxiliary-site geometry

Once a clinically informative, recoverable region is selected, guide activity, CpG position and strand, fragment endpoints, PAM, conversion compatibility, and auxiliary sites should be co-optimized. Restriction-site position can determine post-digestion Cas activation (van Dongen et al., 2021), and native 5 mC effects are themselves geometry-dependent (Yang et al., 2025). PAM-relaxed variants, engineered toeholds, sticky-end substrates, artificial-PAM amplicons, and PAM-free ssDNA triggers expand accessible sequence space (Kleinstiver et al., 2019; Zhang W. et al., 2022; Zhou et al., 2022a; Bagheri et al., 2024; Zhuang et al., 2024; Hu et al., 2026; Zhou et al., 2026). These approaches should adapt CRISPR to validated biomarkers rather than prioritize less informative loci solely for geometric convenience.

### 6.3 Amplification and preservation of methylation information

Amplification can increase analytical sensitivity but erases native methylation information if it precedes stable state encoding. PCR copies nucleotide sequence rather than preserving the original cytosine modification as an independently readable feature (Herman et al., 1996). Amplification is therefore appropriate only after discrimination has been encoded into sequence, fragment integrity, a ligated circle, a released trigger, or another stable transducer. This ordering is used across conversion-, nuclease-gated-, padlock-, and trigger-generating workflows (Li et al., 2019; Wang et al., 2021; Zhou et al., 2022a; Zhou et al., 2022b; Han et al., 2023; Lu et al., 2024; Zhuang et al., 2024; Xie et al., 2026; Zhou et al., 2026).

Ultralow LODs are easier to interpret when state discrimination, nucleic-acid amplification, Cas catalytic gain, and downstream optical or electronic gain are considered separately. DNA/silver nanocluster (AgNC), iontronic, DNAzyme/nanozyme, and vertical organic electrochemical transistor (vOECT) implementations illustrate distinct routes to large output signals (Fan et al., 2026; Ling et al., 2026; Xu et al., 2027). “Amplification-free” should refer specifically to the absence of nucleic-acid copy amplification, not to the absence of Cas collateral cleavage or device-level gain. Downstream gain cannot compensate for an informative target molecule that was never sampled.

### 6.4 Compact multiplex and regional methylation encoding

Compact panel development must test what is lost when a sequencing signature is reduced. Candidate loci should be selected in development data, with feature selection and threshold fitting separated from independent evaluation. The reduced panel should be compared with the parent signature and an appropriate orthogonal assay in the same intended-use population. Relevant endpoints include sensitivity at a fixed specificity, stage and cancer-type coverage, tissue localization when required, and longitudinal detectability for MRD. A high discovery-cohort accuracy does not establish that a small panel retains those properties (Klein et al., 2021; Guo et al., 2017; Mo et al., 2023a). The comparison should also account for fragment recovery and the number of molecules available at each locus.

Each added locus should address a defined biological failure mode, but multiplexing creates coupled analytical costs. Guides can compete for Cas or target access. Collateral-cleavage reporters can cause channel crosstalk, and amplification efficiencies can differ across loci. Signal separation must be demonstrated under high single-target inputs and low mixed-target inputs, not only in singleplex reactions. Each locus requires calibration, and the combined decision rule requires its own validation. Under an illustrative independent any-positive rule with per-locus false-positive probability p and n loci, the panel false-positive probability is 1 − (1 − p)^n^. Correlated errors or a fitted multivariate rule change that expression, so measured panel-level specificity is essential. Partitioning or orthogonal channels may reduce interference, but splitting limited cfDNA among reactions reduces copies per measurement.

Regional methylation may carry more information than isolated CpGs, but it is technically harder to preserve. An assay that responds only when a defined combination of CpGs occurs on the same fragment would retain haplotype information, whereas independent single-CpG measurements would not. Among the CRISPR platforms identified in this review, none has established quantitative single-fragment regional methylation decoding at clinical scale. This remains a specific research objective rather than a current capability.

### 6.5 Readout engineering, device integration, and sample-to-answer deployment

Readouts include fluorescence, lateral flow, nanoparticle-enhanced optics, ratiometric nucleic acids, iontronic and electrochemical interfaces, and organic electrochemical transistors (Wang et al., 2021; Li et al., 2024; Tian et al., 2025; Fan et al., 2026; Ling et al., 2026; Tian et al., 2026; Wang X. et al., 2026; Xie et al., 2026; Xu et al., 2027). Comparison should focus on quantitative capability, background floor, dynamic range, operator dependence, manufacturability, and integration rather than endpoint amplitude. A sequencing-free, on-chip enzymatic cfDNA platform provides a non-CRISPR comparator and has been evaluated for integrated methylation testing in a patient cohort. Broader diagnostic claims still require appropriate benign-disease controls and independent evaluation (Agbarya et al., 2026).

Each readout introduces different implementation requirements. Fluorescence supports kinetic measurement and quantitative calibration but requires controlled excitation, optical detection, and signal normalization. Lateral-flow formats simplify instrumentation and user interpretation but are less naturally suited to precise methylation-fraction quantification. Electrochemical, iontronic, and organic-transistor platforms offer routes to compact electronic readout and device integration (Fan et al., 2026; Xu et al., 2027). They also require reproducible surface functionalization and electrode or nanochannel fabrication, together with control of fouling, signal drift, reference calibration, and scalable manufacturing. These hardware and calibration requirements must be addressed during sample-to-answer integration.

Point-of-care translation also depends on how upstream molecular processing is integrated with the sensor. Most CRISPR methylation workflows combine plasma processing or cfDNA extraction with methylation conversion or nuclease gating, amplification or transduction, and readout. These operations can be performed on the bench, but reproducing the complete workflow in decentralized settings is difficult. Closed cartridges, microfluidic handling, or otherwise integrated workflows could reduce transfer-associated contamination and molecule loss, but only if they preserve recovery of rare informative fragments. Practical evaluation should therefore include hands-on steps, sample-to-answer time, reagent storage and stabilization, temperature-control requirements, failure rate, reader complexity, and batch-to-batch device reproducibility in addition to analytical LOD (Kerachian et al., 2021; Harlé et al., 2026).

A recent approach introduced droplet partitioning into CRISPR methylation sensing by combining MSRE digestion, RPA, and Cas12a in vortex-generated droplets, with reported sensitivity of 5 copies/μL and 0.01% methylation-fraction discrimination in controlled materials (Huang et al., 2026). This remains a partitioning proof-of-concept rather than absolute digital quantification in patient cfDNA. Digital methylation-specific PCR is the more mature low-copy comparator (Zhao et al., 2023; Do Canto et al., 2026). Future digital CRISPR studies should report partition occupancy, false-positive compartments, calibration, and end-to-end recovery. Closed or automated sample-to-answer handling is also relevant because each manual transfer adds contamination and molecular-loss risk (Kerachian et al., 2021).

### 6.6 Controls, quantification, and deployability framework

Controls need to interrogate each stage of the information flow. Fully methylated and unmethylated materials test the primary discriminator. Methylation-fraction gradients test quantitative separation, fragment-matched low-copy standards probe geometry and stochasticity, and large unmethylated excesses challenge leakage. Additional controls should address 5hmC or other modifications, matrix inhibition, and general Cas suppression. Complete-workflow validation must define nominal methylation fractions, limits of blank/detection/quantification, precision, interference, specimen stability, input requirements, and failure criteria rather than infer them from isolated enzyme steps (Yu et al., 2015; Deveson et al., 2021; Kerachian et al., 2021; Alexander et al., 2023; Lockwood et al., 2023; Kresse et al., 2025; Harlé et al., 2026).

A practical appraisal can consider eight dimensions, including clinical relevance, biological specificity, cfDNA recoverability, CRISPR accessibility, quantifiability, workflow complexity, device and manufacturing readiness, and evidence maturity. Their relative importance depends on intended use rather than on a single weighted score. Screening places a premium on very high specificity and manageable downstream workup. MRD or monitoring emphasizes longitudinal low-copy reproducibility, whereas targeted triage or high-risk surveillance may tolerate different throughput and turnaround constraints. Supplementary Table S4 translates these dimensions into an intended-use-oriented biosensor deployability checklist.

## 7 From analytical performance to clinical evidence

### 7.1 Analytical LOD versus clinical sensitivity

An analytical concentration LOD describes detection under a specified material, matrix, volume, replicate design, and decision rule. Many very low reported CRISPR methylation LODs were established using synthetic targets or spiked matrices with amplification or downstream signal gain, rather than fragmented patient cfDNA (Wang et al., 2021; Lu et al., 2024; Yang et al., 2025; Wang X. et al., 2026). These values cannot be extrapolated directly to clinical sensitivity. Workflow loss, endogenous background methylation, locus recovery, and clinical thresholds remain additional constraints. Table 4 places representative values alongside specimen and input information. It is a descriptive comparison, not a performance ranking.

**TABLE 4:** Representative CRISPR methylation platforms with analytical conditions and specimen evidence.

Platform/route	Reported concentration LOD or boundary	Lowest reported discriminable fraction	Material and input for analytical testing	Biological specimen/patient input	Readout	Patient-derived cfDNA?	A/B
Hu et al. (2026)/I	A concentration LOD was not extracted; reported percentage is a fraction metric	0.01% (four-CpG targets); 0.1% (single CpG)	Defined methylated/unmethylated DNA standards: 0.2 μg total before bisulfite conversion	Serum circulating DNA: 29 HCC and 11 ovarian-cancer cases; 100 ng before conversion. Four healthy comparators were leukocyte DNA.	Fluorescence after conversion/PCR and toe-dsDNA activation	Yes; serum circulating DNA.	A5/B1
HGRC, Lu et al. (2024)/II	100 a.m. (defined target)	0.1%	Defined target mixtures: 100 fM total target for fraction testing. HhaI digestion uses 20 μL target in 25 μL; integrated RPA-Cas12a uses 6.6 μL template in 60 μL (Methods)	Cell-line genomic DNA; no patient-plasma experiment identified	Fluorescence after HhaI gating and glycerol-assisted RPA/Cas12a	No.	A3/not demonstrated
Nanoporous-gold assay, Wang et al. (2026a)/II	3.4, 5.2, 4.6 fM for RASSF1A, APC, FOXA1	1%	Synthetic cfDNA-like fragments and artificial plasma. Sample input volume and total reaction volume are not specified in the published Methods; no molecule-count conversion is made	105 blinded contrived specimens; no patient sample input	Square-wave voltammetry on nanoporous gold after HpaII/RPA/Cas12a	No; artificial matrix	A4/not demonstrated
CRISPR-MeDNA, Yang et al. (2025)/IV	∼100 fM (two-guide discrimination); ∼10 pM (single guide)	1% at 1 nM total target.	Synthetic CCDC140 targets; plasma spike/extraction experiments. 2 μL sample in 20 μL reaction	Plasma cfDNA: 15 healthy, 9 lung-cancer and 10 pancreatic-cancer participants; 4 mL plasma, ∼2 ng per 20 μL reaction	Fluorescence inhibition; no nucleic-acid preamplification	Yes; plasma cfDNA.	A5/B1
DL-RLAMP/Cas12a, Zhou et al. (2026)/II	0.18 fM (calculated at signal-to-noise ratio 3)	0.1% at 200 pM total target.	Defined p15 targets. Digestion-ligation volume 10 μL; 4 μL amplified product enters Cas12a readout. Amplicon input is not original cfDNA input	Targets spiked into a 10% serum-mimicking albumin matrix; no patient-derived plasma experiment	Fluorescence after digestion-ligation, RLAMP and Cas12a trans-cleavage	No; surrogate matrix	A4/not demonstrated

Values describe the cited experimental conditions and do not define equivalent clinical sensitivity. A missing or unverified input is not inferred from concentration. “Not demonstrated” means no B-level clinical evidence for the cfDNA, intended use; it does not deny other biological experiments.

Clinical data illustrate the distinction. SEPT9 sensitivity in prospective asymptomatic screening was substantially lower than analytical detectability alone would suggest (Church et al., 2014; Potter et al., 2014), and MCED sensitivity remains strongly stage-dependent despite high specificity (Liu et al., 2020; Klein et al., 2021; Alexander et al., 2023). Results from selected high-risk liver cohorts likewise cannot be generalized to average-risk screening (Zhang et al., 2026). Clinical sensitivity is determined by the complete biological and diagnostic workflow rather than by reporter performance alone.

### 7.2 Molecule availability and stochastic sampling

Let N0 be the expected number of target-locus copies in the starting plasma aliquot, before extraction, reaction allocation, and losses associated with the required assay geometry. It is a locus-copy count, not the number of DNA fragments inferred directly from total cfDNA mass. Let TL be the tumor-derived fraction of those copies. MT and MB are the proportions of tumor-derived and non-tumor copies, respectively, carrying the specified methylation state. Rf is the probability that a molecule retains all required target elements on an interrogable fragment. RW is the fraction of such geometry-compatible molecules recovered into the tested reaction through extraction, processing, and aliquot allocation. All fractions are dimensionless and lie between zero and one. N0, λT, and λB are expected copy counts.

With shared recoverability for the tumor and background populations, λT = N0 × RW × Rf × TL × MT and λB = N0 × RW × Rf × (1 − TL) × MB. Rf and RW must have non-overlapping operational definitions. If measured end-to-end recovery already includes loss of target geometry, that loss must not be counted a second time. The simple product also assumes that the stated recovery factors apply to molecules carrying the specified methylation state. Independence of separately estimated marginal factors should not be presumed.

Under a Poisson approximation for random rare-molecule sampling, P(at least one informative tumor-derived molecule) = 1 − e^−λT^. Thus λ_T_ = 1 gives 63.2%, and λ_T_ ≈ 3 gives 95%. These are sampling probabilities, not diagnostic sensitivities. A real assay additionally depends on λ_B_, assay leakage, recovery uncertainty, and its decision rule.

The shared Rf assumption can fail when tumor and background fragment distributions differ. Separate Rf,T/Rf,B and RW,T/RW,B terms are needed when geometry or processing recovery differs by origin. State-dependent recovery may require further stratification. Copy-number variation changes locus-copy representation and TL. Correlated fragments, heterogeneous sampling rates, batch effects, or nonrandom loss can invalidate the simple Poisson approximation. The model is a bookkeeping aid rather than a validated clinical classifier (Bettegowda et al., 2014; Underhill et al., 2016; Mouliere et al., 2018; Gamisch, 2026; Vijayan Selvarajan et al., 2026). Figure 6 shows the shared-factor special case.

**FIGURE 6:**
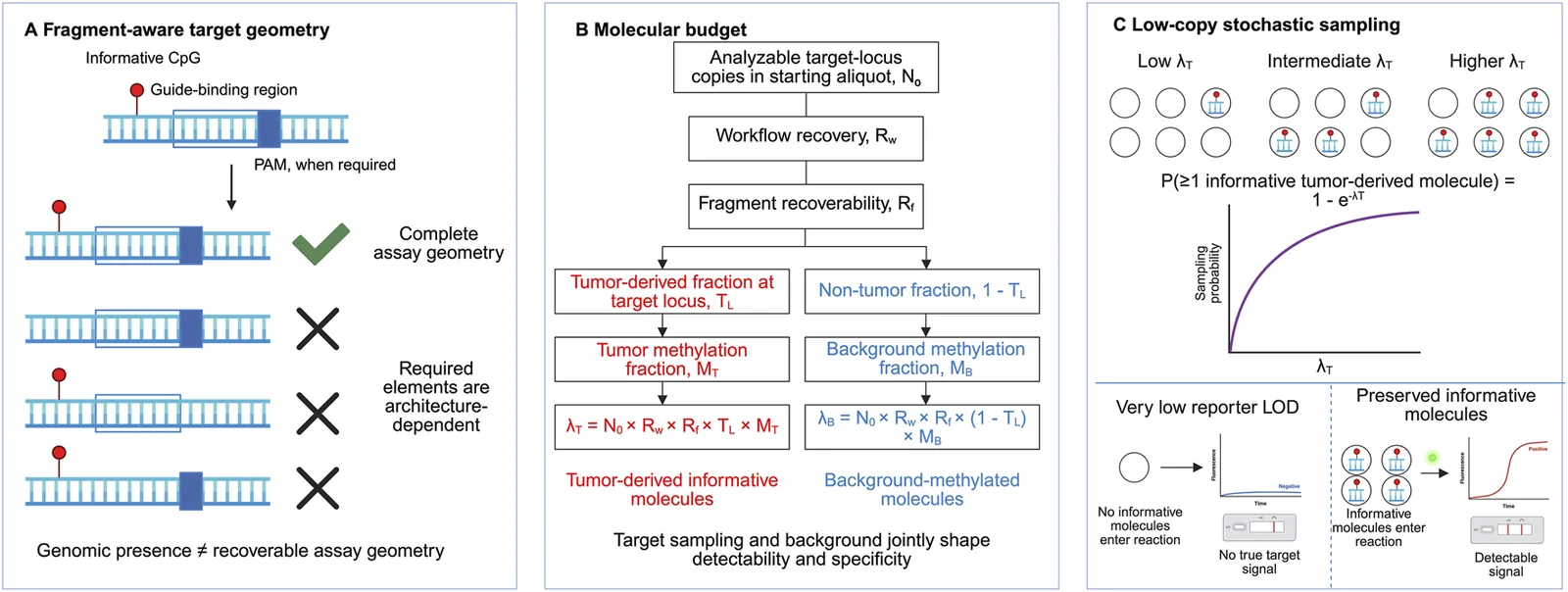
Fragment recoverability and molecular sampling constrain low-copy cfDNA methylation detection. **(A)** Productive interrogation requires informative CpGs and architecture-specific assay elements to coexist on a recoverable cfDNA fragment. **(B)** Molecular-budget framework for the expected numbers of tumor-derived informative molecules (λ_T_) and background-methylated molecules (λ_B_), incorporating starting target-locus copies (N_0_), workflow recovery (R_W_), fragment recoverability (R_f_), locus-specific tumor-derived fraction (T_L_), and tumor and background methylation fractions (M_T_ and M_B_). **(C)** Under Poisson sampling, P(≥1 informative tumor-derived molecule) = 1 − e^−λT^, illustrating why reporter sensitivity cannot compensate when no informative molecule enters the reaction. Abbreviations: cfDNA, cell-free DNA; LOD, limit of detection; PAM, protospacer-adjacent motif. Additional definitions: CRISPR, clustered regularly interspaced short palindromic repeats; Cas, CRISPR-associated protein; CpG, cytosine-phosphate-guanine dinucleotide; DNA, deoxyribonucleic acid. N_0_ is the expected starting locus-copy count before workflow and geometry loss; T_L_ is its tumor-derived fraction. M_T_/M_B_ specify tumor/background methylation fractions, R_f_ geometry compatibility, and R_W_ recovery into the tested reaction conditional on compatible geometry. λ_T_/λ_B_ are expected informative tumor/background copy counts. The shared-factor special case assumes equal tumor/background recovery; geometry loss must not be counted in both R_f_ and R_W_. The Poisson probability is a sampling probability and is not clinical sensitivity.

The measurement boundary for the molecular budget should be stated explicitly. Plasma volume, total extracted cfDNA mass, the aliquot allocated to each reaction, and locus-specific copy availability describe different stages. Total cfDNA mass alone cannot establish the number of informative methylated target molecules. The minimum reporting checklist in Section 7.8 links these inputs to recovery and analytical performance.

### 7.3 Methylation-fraction discrimination and quantitative readout

Concentration LOD and methylation-fraction discrimination answer different questions. Methylation fraction is the fraction of target molecules carrying the specified state and is distinct from tumor fraction or ctDNA fraction. A platform can detect a low concentration of fully methylated DNA yet fail to resolve rare methylated molecules in a large unmethylated background if leakage dominates. Reported CRISPR methylation-fraction values arise from different matrices, inputs, decision rules, and amplification schemes (Wang et al., 2021; Lu et al., 2024; Huang et al., 2026; Li et al., 2026b; Xie et al., 2026; Zhou et al., 2026). We therefore describe the “lowest reported discriminable methylation fraction” rather than a standardized minimum detectable fraction.

Quantitative clinical assays show why calibration choices matter. In the evaluated colorectal-cancer surveillance population, applying a reporting threshold for methylated BCAT1/IKZF1 yielded higher recurrence specificity and positive predictive value with little sensitivity loss (Pedersen et al., 2022). Digital methylation-specific PCR enables Poisson-based absolute quantification at low copy numbers, although its effective LOD remains constrained by sample input, partition occupancy, recovery, and false-positive partitions (Zhao et al., 2023; Do Canto et al., 2026). MCED analytical validation similarly distinguishes limit-of-detection experiments from clinical classification thresholds (Alexander et al., 2023). Future CRISPR studies should report limit of blank, limit of detection, limit of quantification when meaningful, dynamic range, run-to-run coefficient of variation, and the lowest discriminable methylation fraction in a realistic excess of unmethylated target molecules.

For native inhibition readouts, low fluorescence can reflect methylation, insufficient target, matrix inhibition, nuclease instability, or inaccessible guide geometry. The paired-control strategy and invalid-result criteria described in Section 5.5 are therefore necessary parts of quantitative calibration. A ratio does not by itself establish methylation specificity or correct low-copy sampling error.

### 7.4 Four separable dimensions of specificity

Specificity has four separable dimensions. Sequence specificity concerns recognition of the intended locus. Epigenetic-state specificity concerns discrimination between methylated and unmethylated targets. Disease specificity asks whether the measured state is uncommon in relevant non-cancer conditions. Tissue-of-origin specificity concerns assignment of the signal to the clinically relevant organ or cell type. Tissue localization is essential for some MCED applications but may be unnecessary for organ-specific surveillance or patient-specific MRD.

CRISPR assays usually emphasize sequence and epigenetic-state specificity, whereas clinical methylation diagnostics show that disease and tissue specificity can be equally decisive. Cell-type deconvolution defines the structured normal background (Sun et al., 2015; Lehmann-Werman et al., 2016; Moss et al., 2018), and targeted-marker studies show the need for clinically relevant control populations (Kneip et al., 2011; Oh et al., 2013; Church et al., 2014; Pedersen et al., 2015; Park et al., 2026). CancerLocator and MCED classifiers add the separate challenge of tissue localization (Kang et al., 2017; Liu et al., 2020; Klein et al., 2021). Near-perfect molecular discrimination can still fail clinically if the biomarker is nonspecific to disease or tissue context.

### 7.5 A proposed two-axis translational evidence framework

We use two axes to distinguish specimen realism from clinical evidence. Axis A runs from A1 synthetic targets to A5 patient-derived cfDNA and describes the material tested, not overall assay quality. A3 denotes endogenous biological DNA, while A4 adds biofluid-matrix realism with spiked or surrogate targets. Neither automatically outranks the other in every respect. Axis B describes clinical evidence for the cfDNA intended use, from B1 exploratory patient association to B5 implementation. An exploratory tissue or exfoliated-cell study does not establish a B level for a plasma-cfDNA application. Table 3 defines the categories, and Supplementary Table S2 documents the specimen and study-design evidence supporting each A/B assignment.

These categories are proposed conceptual benchmarks, not validated risk-of-bias scores or formal evidence grades for clinical guidelines. Assignment involves expert judgment, and different assessors may classify incompletely reported studies differently. Independent duplicate A/B rating was not performed; hence, Cohen’s kappa was not calculated. Recording the decisive specimen and study-design evidence makes the appraisal auditable, but does not establish its reproducibility. This distinction is consistent with guidance separating analytical validity, clinical validity, utility, and implementation (Pascual et al., 2022; Lockwood et al., 2023; Harlé et al., 2026; Rubinstein et al., 2024).

For B2 or B3, a locked assay has fixed reagent composition, guide/panel selection, processing protocol, instrumentation and analysis settings, normalization, quality-control rules, and decision threshold before testing the validation cohort. Blinding and a prespecified analysis plan help preserve the separation between assay development and validation. Parameters cannot be retuned using validation outcomes and still count as an independent test of the original assay. Any such modification constitutes further development and requires evaluation in a new independent cohort.

### 7.6 Current evidence landscape of published CRISPR methylation assays

The reviewed platforms have progressed further in molecular testing than in clinical validation. Conversion and enzymatic-gating systems cover multiple specimen categories, with relatively few studies using patient-derived cfDNA. CRISPR-MeDNA reaches A5 for specimen type but only B1 for clinical evidence (Yang et al., 2025). Other exploratory circulating-DNA examples include Hu et al. (2026), Fan et al. (2026), and Hou et al. (2026). Table 4 compares selected platforms without equating their LODs, and Supplementary Table S2 provides the broader study inventory and assignment rationales.

None of the representative CRISPR-enabled methylation assays identified here has yet reached B2 validation of a locked assay in an independent cohort with a prespecified decision rule and appropriate comparator. Engineered transduction can strengthen an upstream route but should not be counted as an independent source of methylation specificity.

### 7.7 Candidate clinical settings for research evaluation

Clinical use cases differ in prevalence, required specificity, acceptable workflow, and the value of longitudinal information. Population screening imposes especially stringent specificity and verification requirements because low prevalence increases the burden of false-positive follow-up (Rubinstein et al., 2024). Prospective SEPT9 and community ColonAiQ data illustrate the effects of prevalence, follow-up adherence, and reference-standard verification (Church et al., 2014; Wang et al., 2024). The cited observational MCED evaluations reported high specificity but marked stage dependence (Klein et al., 2021; Gao et al., 2023), while asymptomatic and high-risk cohorts show that performance is population-specific (Nguyen et al., 2025; Zhang et al., 2026; Zhu et al., 2026). Accuracy alone does not establish net clinical benefit.

Candidate near-term settings for targeted adjunctive diagnosis include indeterminate pulmonary nodules, HCC surveillance in high-risk liver disease, and triage after a positive conventional screen. Because these settings involve higher pretest probabilities, they may be more suitable for initial evaluation of compact methylation panels. These remain proposed indications rather than validated CRISPR uses. Relevant benchmarks include plasma SHOX2 and multiplex digital lung methylation assays (Kneip et al., 2011; Zhao et al., 2023), targeted HCC methylation studies (Park et al., 2026; Zhang et al., 2026), and compact gastrointestinal assays (Jiang et al., 2026; Zhu et al., 2026).

Monitoring and MRD are candidates for targeted-assay research because serial testing can exploit within-patient changes and a known disease context. Non-CRISPR methylation studies provide benchmarks for postoperative and longitudinal assessment (Jin et al., 2021; Pedersen et al., 2022; Symonds et al., 2022; Mo et al., 2023b; Nakamura et al., 2026). Associations between postoperative methylation signals and recurrence support prognostic evaluation. They do not demonstrate that test-guided treatment improves outcomes or validate CRISPR performance in MRD. These applications remain challenging because of very low molecule availability, the need for marker stability during treatment, and cumulative false-positive risk across repeated tests. MRD feasibility studies may begin early in development, but routine clinical integration requires the later validation and utility milestones described in the Conclusions.

These clinical settings impose different design priorities. MRD and longitudinal monitoring require reliable molecule recovery and quantitative repeatability at very low target copy number. Stable within-patient baselines are also important. Adjunctive diagnosis and triage place greater emphasis on turnaround time, specificity, and integration with an existing diagnostic pathway. Repeated high-risk surveillance requires sustained specificity across serial testing, whereas population-wide screening adds the most stringent demands for false-positive control, workflow scalability, and independent prospective validation. The optimal CRISPR architecture is therefore likely to depend on intended use rather than on a single analytical performance metric.

Longitudinal methylation sensing may also support treatment-response assessment by tracking molecular change during or after therapy. However, the CRISPR methylation studies reviewed here have not established treatment-guided intervention or real-time therapeutic decision making. These applications should therefore be viewed as a future extension of diagnostic monitoring rather than demonstrated theranostics.

### 7.8 Comparator methods, cohort design, and minimum reporting

A CRISPR methylation assay should be compared with a method that answers the same biological question. Suitable comparators include quantitative methylation-specific PCR (qMSP) or methylation-specific ddPCR for targeted loci, targeted bisulfite or enzymatic sequencing for methylation fraction, and an established clinical assay when available. Digital methylation PCR is especially useful because it provides low-copy molecule counts, while cross-platform studies can reveal hidden variance (Deveson et al., 2021; Lockwood et al., 2023; Zhao et al., 2023; Do Canto et al., 2026).

A non-CRISPR electrical comparator, Asima Rev, measured cfDNA aggregation associated with methylation state in 46 untreated CRC patients and 33 controls (Kaur et al., 2026). Four samples overlapped with the threshold-development cohort, limiting validation independence. Its retrospective case-control performance does not establish population-screening utility. Because this global readout differs from locus-specific methylation measurement, it is not an interchangeable comparator for targeted CRISPR assays.

Patient studies should report intended use, enrollment setting, stage or clinical-state distribution, relevant non-cancer controls, failures, blinding, prespecified cutoffs, confidence intervals, and independence from assay development. Screening studies must also address reference-standard verification. Sensitivity cannot be directly estimated if only test-positive participants receive the reference procedure (Wang et al., 2024).

At minimum, analytical reporting should describe the amount of material entering each stage of the workflow. Required information includes plasma input volume, total extracted cfDNA mass, reaction input mass, sample volume, total reaction volume, allocation across replicates, and measured end-to-end workflow recovery with its operational definition. Studies should also report fragment-matched reference standards, limit of blank, concentration LOD, limit of quantification when applicable, the lowest discriminable methylation fraction at a stated total target input, and within-run and run-to-run precision. Calibration, replicate and calling rules, interference, and invalid-result rates should accompany these metrics. A quantity that was not measured should be identified as such, rather than inferred from reporter brightness or nominal DNA input (Alexander et al., 2023; Lockwood et al., 2023).

Claims from small proof-of-concept cohorts should remain proportional to the evidence. The patient-plasma comparison reported by Fan et al. (2026) supports exploratory group discrimination. Hou et al. (2026) reported 100% sensitivity and specificity in an exploratory analysis of tumor tissues and paired plasma from 15 clinical patients. These point estimates do not establish independent clinical validity. Five tumor versus five adjacent tissues support a tissue-level difference in the tested samples, without estimating liquid-biopsy sensitivity (Xie et al., 2026). Such comparisons do not validate a classifier in other populations. Statistical significance alone is not diagnostic accuracy, and apparently perfect point estimates in small cohorts retain substantial uncertainty.

### 7.9 Reproducibility, standardization, and regulatory translation

For regulatory translation, variability must be controlled across the complete workflow, from venipuncture through classification. Collection, processing, storage, extraction, fragment assessment, conversion or digestion, amplification, Cas/guide activity, reporter stability, sensor fabrication, surface functionalization where applicable, reader calibration, and threshold algorithms can all contribute variance (Kerachian et al., 2021; Harlé et al., 2026). For bioelectronic platforms, electrode or nanochannel fabrication, surface aging, fouling, and lot-to-lot device variability should be treated as part of analytical validation rather than as downstream engineering details. Controls should be tailored to the assay architecture. Route I requires measures of conversion efficiency and recovery, whereas Route II requires digestion-completeness controls. Affinity-based methods require capture-efficiency controls, engineered circuits require leakage controls, and native-state sensing requires methylation-insensitive reaction controls. Shared traceable reference materials and closed-system automation can then support inter-laboratory comparability and reduce molecular loss (Yu et al., 2015).

Regulatory claims must also be indication-specific. An assay validated for recurrence monitoring cannot be assumed suitable for average-risk screening, and a plasma diagnostic in a high-risk liver cohort cannot be generalized to an unselected population without additional evidence. Regulatory development is likely to be more tractable when the biomarker, comparator, intended use, and evidence stage are aligned from the outset.

### 7.10 Limitations of this review

This targeted narrative review is vulnerable to selection bias. Searching PubMed/MEDLINE and Web of Science Core Collection, supplemented by citation tracking, publisher checks, and DOI verification, reduces dependence on any single information source but cannot guarantee comprehensive coverage. Query adaptation and relevance judgments may overrepresent familiar methods, well-cited papers, or positive results. The documented search strategy is intended for a targeted narrative review rather than PRISMA-style exhaustive screening, and no formal risk-of-bias assessment or quantitative weighting was applied. Heterogeneous materials, LOD definitions, amplification schemes, matrices, and reporting prevent a formal cross-platform ranking. The A/B categories are unvalidated expert appraisals, not risk-of-bias grades. Independent duplicate ratings were not performed, so inter-rater reliability is unknown. Future studies should test independent ratings using prespecified rules and examine construct validity alongside design-appropriate tools (e.g., QUADAS-3 for eligible diagnostic accuracy studies; Whiting et al., 2026), recognizing that these assess bias and applicability rather than A/B specimen realism and clinical evidence maturity. Comparative conclusions are limited to the studies identified under the stated review framework. Relevant null or differing findings are discussed, but the search and appraisal cannot establish that all contradictory evidence was captured. We do not pool results across incompatible models, specimens, or clinical designs.

## 8 Outlook

### 8.1 Priority experiments for native-state and regional sensing

Native-state and regional sensing require distinct next-step experiments. Native-state modification sensitivity should be mapped systematically across clinically validated loci, Cas orthologs, guide registers, strands, CpG densities, and competing modifications such as 5hmC, with paired methylation-insensitive controls (Cui et al., 2020; Yang et al., 2025; Roth et al., 2026). The goal is a reproducible methylation-response function rather than maximal nuclease activity. Regional sensing should be tested on cfDNA-like fragments carrying validated co-methylation patterns to determine whether a CRISPR architecture can quantitatively preserve single-fragment haplotype information (Guo et al., 2017; Mo et al., 2023a).

### 8.2 Molecule-aware benchmarking and quantitative validation

Molecule-aware benchmarking should proceed in parallel with mechanistic development. Studies should report plasma input, end-to-end recovery, locus-specific fragment availability, expected informative copy number, limits of blank/detection/quantification where appropriate, lowest discriminable methylation fraction, precision, failure rate, and internal-control behavior (Yu et al., 2015; Deveson et al., 2021; Alexander et al., 2023; Lockwood et al., 2023; Gamisch, 2026; Vijayan Selvarajan et al., 2026). A more informative benchmark is reproducible performance after accounting for the starting molecule budget, fragmentation, and matrix effects. Cross-laboratory and lot-to-lot reproducibility should precede claims of point-of-care or clinical readiness.

### 8.3 Toward integrated biosensor and point-of-care platforms

Moving from molecular assays to deployable biosensors will require integration of cfDNA preparation, methylation discrimination, amplification or transduction, and readout without sacrificing recovery of rare informative molecules. Among the studies identified here, most CRISPR methylation platforms remain modular benchtop workflows rather than fully integrated sample-to-answer systems evaluated with patient-derived cfDNA. For optical and lateral-flow formats, priorities include reagent stabilization, contamination control, quantitative calibration, and simplified thermal handling. For electrochemical, iontronic, and transistor-based platforms, reproducible surface fabrication, fouling resistance, drift correction, reference calibration, and scalable reader or cartridge design become equally important. Patient-plasma studies should therefore report not only analytical sensitivity but also sample-to-answer time, hands-on steps, failure rate, calibration stability, device batch variability, and compatibility with closed or portable workflows. Analytical performance obtained from separately optimized modules cannot be assumed to persist after integration into a closed device.

### 8.4 From validated methylome signatures to intended-use deployment

A compact CRISPR panel derived from a validated methylome signature should be tested as a new assay, with independent evaluation of information loss, fragment recovery, calibration, and panel-level specificity. High-dimensional methylomics remains suited to discovery and tissue-of-origin modeling (Liu et al., 2018; Shen et al., 2018; Klein et al., 2021; Gao et al., 2023; Mo et al., 2023a; Zeng et al., 2026). The choice of an initial use case should be guided by a target product profile and an achievable molecular budget. Higher pretest probability or serial sampling may support feasibility studies, but does not remove the need for intended-use validation and evidence of clinical benefit.

## 9 Conclusion

Preserving informative methylation states from sampling through readout is the central translational challenge. Conversion, enzymatic gating, affinity recognition, and native-state modulation introduce different losses and calibration requirements. Engineered transduction can expand targetability, but cannot recover information lost upstream. Native-state sensing remains a research direction with configuration-dependent *in vitro* findings and exploratory observational evidence from patient-derived cfDNA. No native-state Cas methylation assay identified here has established clinically usable diagnostic performance.

Near-term priorities include harmonized reporting of LOD, methylation fraction, input, recovery, and precision, together with fragment-matched reference materials and small, well-controlled feasibility studies using patient-derived cfDNA and orthogonal comparators. Native-state studies additionally need the inhibition and modification controls specified in Section 5.5. Mid-term priorities are independent validation of compact multiplex panels with locked protocols and thresholds, clinically relevant benign/inflammatory controls, and demonstrated panel-level specificity. Closed sample-to-answer devices should be assessed for recovery, failure rate, calibration stability, and manufacturing reproducibility. Intended use and the evidence required for the applicable regulatory pathway should be defined during this stage. Long-term clinical integration into MRD monitoring, high-risk surveillance, or other care pathways requires prospective intended-use evaluation and evidence that test-guided management provides clinical benefit. These are evidence milestones rather than fixed timelines. Targeted CRISPR may complement high-dimensional methylomics if compact signatures retain sufficient clinical information and reproducibility in patient plasma.
