# Supplementary material for: CRISPR-enabled biosensing of cancer-associated DNA methylation in cell-free DNA: molecular architectures, molecular sampling constraints, and translational biosensor engineering
Source: Front Bioeng Biotechnol. 2026 Oct 27;14:1986098. doi: 10.3389/fbioe.2026.1986098 (PMC13747777; doi:10.3389/fbioe.2026.1986098)
Supplement: Supplementary file 1 [file DataSheet1.pdf]

Supplementary Material

1 Supplementary Tables

The supplementary tables provide methodological and study-level detail that is impractical to include in the main article. Supplementary Table S1 documents literature identification, study selection, and evidence classification. Supplementary Table S2 is representative rather than exhaustive and summarizes CRISPR-enabled methylation sensing studies by primary methylation-discrimination route, with molecular/specimen evidence and clinical evidence maturity reported separately. Supplementary Table S3 compiles selected clinical cfDNA methylation benchmarks, and Supplementary Table S4 provides a biomarker-first biosensor deployability checklist.

2 Supplementary Note. Literature identification and evidence classification

Literature identification used targeted, non-exhaustive searches of PubMed/MEDLINE and Web of Science Core Collection, supplemented by publisher-site checks, broader bibliographic searches of engineering and biosensor studies, backward and forward citation tracking, and DOI verification. Searches were last updated on 31 August 2026. English-language peer-reviewed publications were eligible, with no lower publication-date limit; preprints and grey literature were excluded. Peer-reviewed online-first articles with permanent DOIs were eligible by the coverage cutoff even when issue assignment occurred later. 5-Hydroxymethylcytosine (5hmC) studies were retained only when they informed modified-base sensing mechanisms, interference, or affinity/transduction precedents and were not treated as equivalent clinical evidence for 5-methylcytosine (5mC) cancer-associated methylation assays. Evidence-stage assignments represent the authors' structured appraisal according to the predefined main-text criteria rather than a validated grading system. Supplementary Table S1 records the databases, final search date, search concepts and query formulations, selection rules, eligibility boundaries, data-extraction fields, and evidence-classification criteria. The recorded query formulations were adapted to database-specific syntax and to the mechanistic or translational question. The A/B framework is conceptual and has not been validated for guideline evidence grading or risk-of-bias assessment. Independent duplicate A/B assignments were not performed; accordingly, inter-rater reliability (including Cohen's kappa) was not estimated. Axis A describes the materials demonstrated, with A3 endogenous biological DNA and A4 spiked/surrogate biofluid experiments representing different facets of specimen realism. A5 requires patient-derived circulating DNA. Axis B refers specifically to clinical evidence for a cfDNA intended use; clinical results derived solely from tissue or exfoliated-cell DNA do not establish a B level for that use.

Supplementary Table S1. Literature search, study selection, and evidence-classification framework.

Component	Sources or representative search concepts	Selection or appraisal rule	Use in this review
Review design	Targeted, non-exhaustive mechanistic narrative review focused on CRISPR-enabled methylation sensing, cfDNA biology, biomarker validation, and clinical translation.	Primary studies were prioritized for detailed appraisal. The review was not designed as a systematic review or meta-analysis and did not use PRISMA-style record screening or pooled effect estimation.	Basis for a mechanism- and translation-focused synthesis rather than pooled effect estimation.
Literature coverage and information sources	Final search update: 31 August 2026. No lower publication-date limit. PubMed/MEDLINE and Web of Science Core Collection were the primary bibliographic databases. Publisher websites, DOI verification, backward/forward citation tracking, and targeted engineering/biosensor searches supplemented database retrieval.<br>Final search date: 31 August 2026.	Records were checked for direct relevance, bibliographic accuracy, article type, DOI, and publication status. Engineering, microfluidic, materials, and biosensor terms were searched separately when biomedical indexing was likely to be incomplete.<br>Update verification: Database discovery in PubMed/MEDLINE and Web of Science Core Collection was followed by publisher and DOI verification; a later issue date did not reclassify an earlier online publication as new.	Defines the literature-update window and broadens coverage across biomedical and engineering sources.
Core search concepts	CRISPR/Cas; DNA methylation; 5mC; 5hmC; cell-free DNA/cfDNA; circulating tumor DNA/ctDNA; cancer; liquid biopsy; analytical validation; clinical validation.	Concept blocks were combined according to the mechanistic or clinical question being updated rather than applied as one fixed Boolean string.<br>Scope of documentation: The recorded query formulations document the targeted narrative search. Database field tags and syntax were adapted between PubMed/MEDLINE and Web of Science Core Collection. Because this was not a systematic review, PRISMA-style retrieval counts and record-screening flow were not generated.	Covers direct CRISPR assays, cfDNA biology, methylation biomarkers, and translational comparators.

Component	Sources or representative search concepts	Selection or appraisal rule	Use in this review
CRISPR-enabled methylation sensing	Representative query: ("DNA methylation" OR 5mC OR 5hmC) AND (CRISPR OR Cas12a OR Cas12b OR Cas13a OR Cas9).<br>CRISPR update query: ("DNA methylation" AND (CRISPR OR Cas12a OR Cas13a) AND 2026)	Priority was given to studies in which Cas contributed directly to methylation detection or downstream signal transduction. 5hmC studies were retained only when they informed modified-base sensing mechanisms, interference, or affinity/transduction precedents rather than as equivalent clinical evidence for 5mC cancer-associated methylation assays.	Core CRISPR-enabled methylation evidence base summarized in Supplementary Table S2.
Engineering and biosensor coverage	Representative concepts: DNA methylation AND CRISPR/Cas12a AND (droplet OR digital OR microfluidic OR biosensor OR nanozyme OR transistor OR materials).	Targeted searches were used to identify engineering papers that may not be captured reliably by biomedical database indexing. Studies were retained when the methylation discriminator and Cas contribution could be appraised directly.	Adds microfluidic, materials, device, and signal-transduction studies that may be incompletely indexed in biomedical databases.
Native-state / direct Cas sensing	Representative query: (5mC OR methylation) AND (Cas12a OR Cas9) AND (direct OR native OR inhibition OR modification-sensitive).	Studies were retained when methylation itself modulated Cas-target recognition without upstream methylation conversion or auxiliary methylation discrimination.	Operational definition for Route IV and for distinguishing direct Cas sensing from conversion-free but indirect designs.
Affinity-assisted recognition	Representative query: (MBD OR MeCP2 OR cfMeDIP OR MeDIP OR anti-5mC) AND (cfDNA OR plasma) AND cancer.<br>**Affinity-enrichment query:** ((MBD OR MeCP2 OR cfMeDIP OR MeDIP OR anti-5mC) AND cfDNA AND cancer)	Affinity studies were included when they informed methylated-DNA capture/gating, low-input plasma feasibility, or a credible affinity-to-Cas transduction precedent.	Evidence base for Route III while retaining the limited maturity of direct 5mC affinity-to-Cas sensing in cancer-associated cfDNA.
cfDNA biology and fragmentation	Representative query: (cfDNA OR "cell-free DNA") AND (fragmentation OR fragmentomics OR nucleosome OR recoverability) AND (methylation OR cancer).	Studies were selected when they informed fragment length/end structure, locus recoverability, tumor fraction, molecular sampling, or preanalytical constraints relevant to targeted assays.	Biological basis for fragment-aware assay design and interpretation of analytical sensitivity.

Component	Sources or representative search concepts	Selection or appraisal rule	Use in this review
Cancer-associated methylation biomarkers	Representative query: (cfDNA OR ctDNA OR plasma) AND methylation AND (biomarker OR detection OR tissue-of-origin) AND cancer.<br>Prediagnostic methylation query: (prediagnostic plasma AND methylation AND cancer signal) OR (methylated DNA biomarkers AND preclinical cancer)	Clinically evaluated loci, regional/haplotype markers, and high-dimensional methylation signatures were prioritized when they informed biomarker selection or targeted deployment.	Clinical and biological basis for biomarker-first target selection and comparison with CRISPR deployment.
Clinical validation, screening, and MRD	Representative concepts: methylation AND cfDNA AND (prospective OR validation OR screening OR MCED OR MRD OR recurrence).<br>Multicancer prospective query: (methylation AND fragment size AND multicancer AND prospective asymptomatic)<br>Targeted CRC query: (ctDNA methylation AND colorectal AND prospective AND multi-locus AND 2026)	Prospective or independently validated studies were prioritized when they reported intended-use cohorts, stage-stratified performance, predictive values, longitudinal performance, or follow-up.	Clinical benchmarks summarized in Supplementary Table S3; keeps analytical sensitivity separate from patient-level performance.
Comparator methods and standards	Targeted searches for qMSP and dPCR, methylation sequencing, nanopore or native methylation calling, preanalytical practices, reference materials, and cfDNA analytical-validation guidance.	Non-CRISPR studies, reviews, and guidelines were retained when they supplied essential standards, comparator performance, or mechanistic context not provided by CRISPR studies.	Translational benchmarks and standards used without treating indirect evidence as a substitute for primary CRISPR data.
Core study selection	Primary studies directly informing at least one of four domains: CRISPR-enabled methylation sensing; cfDNA biology/fragmentation; cancer-associated methylation biomarker validation; or analytical/clinical translation.	Direct mechanistic evidence and patient-derived cfDNA evidence were prioritized when available; relevance was judged against the specific claim being supported.	Defines the core evidence base for the recognition/transduction framework and translational analysis.

Component	Sources or representative search concepts	Selection or appraisal rule	Use in this review
Study-level data extraction	For representative CRISPR-enabled studies: target/intended use, first methylation discriminator, primary discrimination route, engineered transduction when present, Cas system, amplification, readout, analytical performance, specimen/cohort, and biological/clinical evidence.	Reported metrics were retained in their original analytical context; concentration LOD, methylation-fraction discrimination, matrix testing, and patient-level validation were not treated as interchangeable.	Source fields for Supplementary Table S2 and the main-text cross-route comparison.
Mechanistic classification	Four primary methylation-discrimination routes: sequence conversion; enzymatic gating; affinity-assisted recognition; and intrinsic/native-state Cas modulation. Engineered molecular transduction is treated as an orthogonal enabling layer.	The primary route was assigned according to where methylated and unmethylated molecules first became distinguishable. Engineered transduction was recorded separately when an upstream discriminator was recoded into an artificial PAM, toehold, ssDNA trigger, or DNA-circuit state.	Separates the origin of methylation specificity from downstream transduction, amplification, and readout.
Translational evidence classification	Two independent axes in main-text Table 3: Axis A for molecular/specimen evidence (A1-A5) and Axis B for clinical evidence maturity (B1-B5).	A/B categories were assigned separately. A3 and A4 distinguish endogenous cell/tissue DNA from spiked/surrogate biofluid testing, not universal quality ranks. A5 requires patient circulating DNA; tissue-only findings do not confer B evidence for cfDNA. These were author appraisals without independent duplicate rating or inter-rater estimates.	Maintains separate molecular/specimen and clinical-evidence positions and prevents specimen realism from being treated as equivalent to clinical validity.
Bibliographic verification	PubMed/MEDLINE and Web of Science Core Collection records, publisher pages, DOI records, exact-title searches, and targeted bibliographic checks for recent or online-first records.	Article type, DOI, publication year, online-publication status, and issue assignment were checked before final citation formatting.	Quality control for article type, DOI, year, online-publication status, and issue assignment.
Eligibility boundaries and verification	English-language peer-reviewed publications relevant to the defined mechanistic or translational question; no lower publication-date limit. Preprints and grey literature were excluded. Articles first available online with a permanent DOI by 31 August 2026 were eligible even when issue assignment occurred later.	Core mechanistic and clinical claims require primary-source support; reviews and guidelines provide context or methods. No formal risk-of-bias instrument or quantitative weighting was used. Evidence categories reflect specimen type and study design rather than a validated grading instrument.	Clarifies eligibility and verification boundaries without implying systematic-review screening or pooled synthesis.

Supplementary Table S2. Representative study-level characteristics of CRISPR-enabled DNA methylation sensing assays, grouped by primary discrimination route.

Study	Target / intended use	Primary methylation discriminator; enabling transduction	Cas, amplification, and readout	Selected analytical performance	Specimen evidence and clinical evidence
Route I — Sequence conversion					
Li et al. (2019)	COL1A2 CpG; methodological proof-of-concept	Bisulfite conversion; Route I	AacCas12b; PCR/LAMP; fluorescence	Methylation-ratio calibration $R^2 \approx 0.9975$; no cfDNA-specific LOD reported	Synthetic targets and cancer-cell gDNA; no patient cfDNA. Main limitation: conversion loss and amplification dependence. Specimen evidence: A3 (Endogenous biological DNA); clinical evidence: none for cfDNA clinical validation.
L. Zhang et al. (2022)	Cancer methylation target model	Bisulfite + methylation-selective padlock ligation; Route I + engineered transduction	Cas12a; RCA + nicking/recycling cascade; fluorescence	LOD 342 aM; lowest reported discriminable methylation fraction ≈0.01%	Synthetic mixtures and cell lysate; no patient cfDNA. Specimen evidence: A3 (Endogenous biological DNA); clinical evidence: none for cfDNA clinical validation.
Han et al. (2023)	p16/CDKN2A; cancer-cell/tissue methylation	Bisulfite conversion + ligation/transcription; Route I + engineered transduction	Cas12a; T7 transcription; single-molecule fluorescence	LOD 337 aM; lowest reported discriminable methylation fraction ≈0.01%	Cancer-cell gDNA and 5 breast cancer versus 4 normal tissues; no plasma cfDNA. Specimen evidence: A3 (Endogenous biological DNA); clinical evidence: none for cfDNA clinical validation.
Li et al. (2024)	Nine-region methylation panel; colorectal cancer model	Bisulfite conversion + multiplex MSP; Route I + engineered transduction	Cas12a; multi-crRNA point/region readout; nanoparticle-stabilized fluorescence	Reported LOD ≈250 aM; lowest reported discriminable methylation fraction ≈0.01%	4 CRC tissue DNA and 16 healthy buffy-coat DNA plus cell lines; no plasma cfDNA. Specimen evidence: A3 (Endogenous biological DNA); clinical evidence: none for cfDNA clinical validation.

Study	Target / intended use	Primary methylation discriminator; enabling transduction	Cas, amplification, and readout	Selected analytical performance	Specimen evidence and clinical evidence
Zhuang et al. (2024)	COL1A2 CpGs	TET2/APOBEC enzymatic conversion; Route I + engineered transduction	Cas12a; PCR; artificial PAM where required; fluorescence/LFA	Fraction-response calibration $R^2 = 0.98$; correlation after preprocessing $R^2 = 0.97$; workflow ≈6 h. A lowest discriminable fraction was not established from the verified main text.	Cell-line gDNA; no patient cfDNA. Enzymatic conversion is state conversion, not native-state sensing. Specimen evidence: A3 (Endogenous biological DNA); clinical evidence: none for cfDNA clinical validation.
Hu et al. (2026)	Multiple cancer methylation loci; HCC/ovarian proof-of-concept	Bisulfite conversion + engineered toe-dsDNA/assist strand; Route I + engineered transduction	Cas12a; PCR; PAM-free downstream activation; fluorescence	Reported methylation-fraction discrimination to ≈0.01% for several targets	Patient-derived circulating DNA from serum of 29 HCC and 11 ovarian-cancer cases; the 4 healthy comparators were leukocyte DNA rather than serum cfDNA controls. No independent validation. Specimen evidence: A5 (Patient-derived cfDNA/circulating DNA) for the cancer-case circulating-DNA specimens; clinical evidence: B1 (Exploratory patient association), without a matched healthy-serum control group.
Route II — Enzymatic gating					

Study	Target / intended use	Primary methylation discriminator; enabling transduction	Cas, amplification, and readout	Selected analytical performance	Specimen evidence and clinical evidence
Wang et al. (2021)	SEPT9; colorectal cancer biomarker model	Dual BstUI/HhaI MSRE gating; Route II	LwCas13a; RPA + T7 transcription; fluorescence/LFA	LOD 86.4 aM; lowest reported discriminable methylation fraction ≈0.01%; visual LFA ≈200 aM	Synthetic/plasmid targets and diluted-serum spike-ins; no patient cfDNA. Extreme LOD depends on multistage amplification. Specimen evidence: A4 (Spiked biofluid/cfDNA surrogate); clinical evidence: none for cfDNA clinical validation.
van Dongen et al. (2021)	MAL single CpG	AciI/MSRE gating; Route II	Cas12a; no major preamplification; fluorescence	Linear methylation-fraction response at 10 nM total DNA (R^2≈0.999); sensitivity inadequate at low input	Synthetic and enzymatically methylated genomic DNA; no patient cfDNA. Fragment geometry affected activity; methylation alone did not significantly change average Cas12a trans-cleavage in the tested configuration. Specimen evidence: A2 (Constructed/reference DNA); clinical evidence: none for cfDNA clinical validation.
Zhou et al. (2022b)	SEPT9; colorectal cancer biomarker model	GlaI methylation-dependent cleavage; Route II	AsCas12a; double-cascade SDA; fluorescence	LOD 1.28×10^-13 M; lowest reported discriminable methylation fraction ≈0.1%; gDNA LOD ≈37.95 ng	Synthetic DNA, serum spike-ins, and HCT116/NCM460 gDNA; no patient cfDNA. Specimen evidence: A4 (Spiked biofluid/cfDNA surrogate); clinical evidence: none for cfDNA clinical validation.

Study	Target / intended use	Primary methylation discriminator; enabling transduction	Cas, amplification, and readout	Selected analytical performance	Specimen evidence and clinical evidence
Zhou et al. (2022a)	RASSF1A; cancer methylation model	HhaI protection plus ssDNA recoding; Route II + engineered transduction	Cas12a; RPA + lambda exonuclease; PAM-free ssDNA activation; fluorescence/LFA	Fluorescence LOD ≈0.98 fM; lowest reported discriminable methylation fraction ≈0.05%; LFA ≈20 fM	Synthetic targets, diluted-serum spike-ins, and cell gDNA; no patient cfDNA. Specimen evidence: A4 (Spiked biofluid/cfDNA surrogate); clinical evidence: none for cfDNA clinical validation.
Wu et al. (2023)	CLDN11; lung adenocarcinoma	GlaI methylation-dependent cleavage; Route II + engineered transduction	Cas12a; EXPAR; fluorescence	LOD ≈1.25 fM; lowest reported discriminable methylation fraction ≈0.1%; serum-spike LOD ≈3.09 fM	Cancer-cell gDNA, serum spike-ins, and 20 lung-adenocarcinoma versus 16 paracancerous tissues; no patient plasma CRISPR classification. Specimen evidence: A4 (Spiked biofluid/cfDNA surrogate); clinical evidence: none for cfDNA clinical validation.

Study	Target / intended use	Primary methylation discriminator; enabling transduction	Cas, amplification, and readout	Selected analytical performance	Specimen evidence and clinical evidence
Xu et al. (2023)	SEPT9; cervical cancer detection	BstUI/MSRE gating; Route II	Cas12a; RPA; fluorescence	≈1 copy/µL; lowest reported discriminable methylation fraction ≈0.01%	53 cervical exfoliated-cell DNA samples: 27/27 cancers positive and 24/26 controls negative (reported sensitivity 100%, specificity 92.3%). Not plasma cfDNA. Specimen evidence: A3 (Endogenous biological DNA); clinical evidence: none for cfDNA clinical validation. The reported cervical exfoliated-cell accuracy is not a validation estimate for a plasma/serum cfDNA application.
Ding et al. (2024)	SEPT9; colorectal cancer model	AciI/MSRE gating; Route II + engineered transduction	Cas12a; CHA; ratiometric FRET fluorescence	LOD 2.02 fM	3 CRC tumor and peripheral-blood gDNA samples versus 3 healthy blood controls; blood material was genomic DNA, not cfDNA. Specimen evidence: A3 (Endogenous biological DNA); clinical evidence: none for cfDNA clinical validation.

Study	Target / intended use	Primary methylation discriminator; enabling transduction	Cas, amplification, and readout	Selected analytical performance	Specimen evidence and clinical evidence
Lu et al. (2024)	CNE2 locus; nasopharyngeal-carcinoma cell model	HhaI/MSRE gating; Route II	Cas12a; glycerol-enhanced phase-separated RPA; fluorescence	LOD 100 aM; 0.1% methylated fraction at 100 fM total target; total analysis ≈70 min. Digestion: 20 µL target in 25 µL; integrated RPA-Cas12a: 6.6 µL template in 60 µL.	Synthetic targets and CNE2/NP69 cell-line gDNA; no patient plasma. Specimen evidence: A3 (Endogenous biological DNA); clinical evidence: none for cfDNA clinical validation.
Pataer et al. (2024)	RARB/RASSF1A methylation models	Single MSRE gating; Route II	Cas12a; cascade exponential amplification; visual fluorescence	Reported ≈1 aM (≈6 molecules/10 µL); lowest reported discriminable methylation fraction ≈0.1%	Cell-line gDNA and spike-in gDNA; no patient plasma. Long digestion and multistage amplification limit sample-to-answer simplicity. Specimen evidence: A3 (Endogenous biological DNA); clinical evidence: none for cfDNA clinical validation.
Ding et al. (2025)	SEPT9; colorectal cancer model	AciI protection or GlaI cleavage; Route II (+ engineered transduction for SDA route)	Cas12a; direct or SDA-generated PAM-free ssDNA; fluorescence	AciI route LOD 0.4414 nM; GlaI-SDA route LOD 8.19 fM	Plasma spike-ins, HCT116/NCM460, and 3 CRC versus 3 healthy tissue/peripheral-blood gDNA. Not patient cfDNA. Specimen evidence: A4 (Spiked biofluid/cfDNA surrogate); clinical evidence: none for cfDNA clinical validation.

Study	Target / intended use	Primary methylation discriminator; enabling transduction	Cas, amplification, and readout	Selected analytical performance	Specimen evidence and clinical evidence
Tian et al. (2025)	SEPT9; colorectal cancer model	AciI/MSRE gating; Route II + engineered transduction	Cas12a; DNAzyme/split-aptamer/Auramine O label-free cascade	Linear 2–200 nM; LOD 1.74 nM; tissue/blood-gDNA CRC discrimination AUC 0.722	Cell lines, paired tissues, and peripheral-blood gDNA; no plasma cfDNA. Main contribution is label-free readout rather than low-copy sensitivity. Specimen evidence: A3 (Endogenous biological DNA); clinical evidence: none for cfDNA clinical validation.
Fan et al. (2026)	SEPT9; colorectal cancer proof-of-concept	AciI/MSRE gating; Route II + engineered transduction	Cas12a + CHA + Ag-DNAzyme + Au/Pt nanozyme; iontronic nanochannel readout	LOD 34.0 aM; rare methylated allele detectable at 1:100 methylated-to-unmethylated ratio	Patient-derived plasma cfDNA from CRC and healthy participants showed exploratory group discrimination. Specimen evidence: A5 (Patient-derived cfDNA); clinical evidence: B1 (exploratory observational patient-level association). Independent validation of a locked assay was not established.

Study	Target / intended use	Primary methylation discriminator; enabling transduction	Cas, amplification, and readout	Selected analytical performance	Specimen evidence and clinical evidence
Hou et al. (2026)	FEZF2 (and OTP) breast cancer methylation	BstUI/MSRE gating; Route II	Cas12a; thermosensitive-hydrogel separated RPA; fluorescence	LOD 1×10^-8 ng/μL; reported methylated fraction 0.05%	Tumor tissues and paired plasma from 15 clinical patients; reported sensitivity and specificity both 100% in this exploratory analysis. Specimen evidence: A5 (Patient-derived cfDNA); clinical evidence: B1 (exploratory observational patient-level association). An independently validated locked assay was not established.
Ling et al. (2026)	Five methylation loci; multigene profiling	HpaII/MSRE gating; Route II + engineered transduction	Cas12a; RPA; cleavage-responsive DNA/Ag nanocluster fluorescence	LOD 74.5 aM with RPA (1.82 fM without RPA); lowest reported discriminable methylation fraction ≈0.01%; five-locus mean LOD ≈140 aM	Serum and cell-lysate models; no patient plasma. Specimen evidence: A4 (Spiked biofluid/cfDNA surrogate); clinical evidence: none for cfDNA clinical validation.
Tian et al. (2026)	SEPT9; colorectal cancer model	AciI/MSRE gating; Route II + engineered transduction	Cas12a; dual-fluorescence functional nucleic-acid ratiometric readout	LOD 152 pM	Cell lines, paired tissues, and 3 CRC versus 3 healthy peripheral-blood gDNA; no plasma cfDNA. Specimen evidence: A3 (Endogenous biological DNA); clinical evidence: none for cfDNA clinical validation.

Study	Target / intended use	Primary methylation discriminator; enabling transduction	Cas, amplification, and readout	Selected analytical performance	Specimen evidence and clinical evidence
Wen et al. (2026)	SEPT9 methylation + KRAS G12C mutation	GlaI methylation gating plus FEN1 mutation discrimination; Route II + engineered transduction	Cas12a; dual SDA inputs; kinetic logic-gate readout	Three kinetic output states demonstrated; no clinically validated LOD/cutoff for multiplex diagnosis	3 CRC versus 3 healthy tumor/peripheral-blood gDNA; no KRAS-G12C-positive clinical case in the small cohort. Specimen evidence: A3 (Endogenous biological DNA); clinical evidence: none for cfDNA clinical validation.
Xie et al. (2026)	SEPT9; colorectal cancer tissue model	GlaI methylation-dependent cleavage; Route II + engineered transduction	Cas12a; double-cascade SDA; G-triplex/ThT fluorescence and LFA	LOD 170.66 fM; lowest reported discriminable methylation fraction ≈0.005%	CRC cell lines and 5 CRC versus 5 adjacent tissues; no plasma cfDNA. Specimen evidence: A3 (Endogenous biological DNA); clinical evidence: none for cfDNA clinical validation.
Zhou et al. (2026)	p15 methylation; methyltransferase activity	Dual HhaI/BstUI gating + padlock circularization; Route II + engineered transduction	Cas12a; rolling circle extension-assisted LAMP (RLAMP) with an engineered PAM-containing activator; fluorescence	LOD 0.18 fM (signal-to-noise ratio 3); 0.1% methylated fraction at 200 pM total target. Digestion-ligation volume 10 µL; 4 µL amplified product enters Cas12a readout.	Defined targets spiked into a 10% serum-mimicking recombinant human serum albumin matrix; no cancer-patient plasma. “One-step” refers to digestion-ligation, followed by amplification and a separate Cas12a reaction. Specimen evidence: A4 (spiked/surrogate matrix); no demonstrated B level for cfDNA.

Study	Target / intended use	Primary methylation discriminator; enabling transduction	Cas, amplification, and readout	Selected analytical performance	Specimen evidence and clinical evidence
Xu et al. (2027)	SEPT9; device proof-of-concept	AciI/MSRE gating; Route II	LbaCas12a; gate-surface ssDNA cleavage; vertical organic electrochemical transistor	LOD 100 fM in buffer; 100 fM–100 pM quantitative range; artificial-serum recoveries ≈91–114%	Artificial serum only; no patient cfDNA. No nucleic-acid preamplification, but substantial device-level signal gain. Specimen evidence: A4 (Spiked biofluid/cfDNA surrogate); clinical evidence: none for cfDNA clinical validation.
Li et al. (2026b)	SEPT9; colorectal cancer biomarker model	Dual HhaI/BstUI MSRE gating + asymmetric RPA; Route II + engineered transduction	Cas12a; asymmetric RPA-generated ssDNA; PAM-independent fluorescence readout	LOD 0.5 fM; lowest reported discriminable methylation fraction ≈1%	Defined DNA materials; no patient cfDNA reported. Specimen evidence: A2 (Constructed/reference DNA); clinical evidence: none for cfDNA clinical validation.
Huang et al. (2026)	FOXD3; semi-digital methylation proof-of-concept	MSRE digestion; Route II + droplet partitioning	Cas12a; RPA; vortex-generated droplets; semi-digital fluorescence	5 copies/μL; lowest reported discriminable methylation fraction ≈0.01%; workflow ≈120 min	Controlled methylated/unmethylated target materials; no patient cfDNA reported. Specimen evidence: A2 (Constructed/reference DNA); clinical evidence: none for cfDNA clinical validation.

Study	Target / intended use	Primary methylation discriminator; enabling transduction	Cas, amplification, and readout	Selected analytical performance	Specimen evidence and clinical evidence
X. Wang et al. (2026)	RASSF1A/APC/FOXA1 three-marker panel; breast-cancer liquid-biopsy model	HpaII/MSRE gating; Route II	Cas12a; RPA; nanoporous-gold electrode with methylene-blue ssDNA reporters; square-wave voltammetry	Target-dependent LODs 3.4, 5.2, and 4.6 fM; 1% methylation-fraction discrimination; artificial-plasma recovery 92.4–106.8%; blinded contrived-specimen AUC 0.966	Synthetic cfDNA-like targets, artificial plasma, and 105 blinded contrived methylation-positive/negative specimens; no patient-derived cfDNA. Specimen evidence: A4 (Spiked biofluid/cfDNA surrogate); clinical evidence: none. The study explicitly frames the work as nonclinical model analysis.
Route IV — Native-state Cas modulation					
Yang et al. (2025)	CCDC140; lung/pancreatic cancer proof-of-concept	Intrinsic 5mC-dependent Cas modulation; Route IV (native-state)	Fn/Lb/AsCas12a; no preamplification; fluorescence inhibition	Two-guide defined-target discrimination ~100 fM; 1% methylated fraction at 1 nM total target.	Patient cfDNA: 15 healthy, 9 lung cancer, and 10 pancreatic cancer; approximately 2 ng cfDNA/reaction from 4 mL plasma; no prespecified cutoff, sensitivity, or specificity estimates. Specimen evidence: A5 (Patient-derived cfDNA); clinical evidence: B1 (exploratory observational patient-level association).

Abbreviations: AgNC, silver nanocluster; AUC, area under the curve; cfDNA, cell-free DNA; CRISPR, clustered regularly interspaced short palindromic repeats; Cas, CRISPR-associated protein; CHA, catalytic hairpin assembly; CRC, colorectal cancer; DCSDA, double-cascade strand-displacement amplification; EXPAR, exponential amplification reaction; FEN1, flap endonuclease 1; FRET, Förster resonance energy transfer; gDNA, genomic DNA; HCC, hepatocellular carcinoma; LAMP, loop-mediated isothermal amplification; LFA, lateral-flow assay; LOD, limit of detection; MSP, methylation-specific PCR; MSRE, methylation-sensitive restriction endonuclease; PAM, protospacer-adjacent motif; PCR, polymerase chain reaction; RCA, rolling-circle amplification; RPA, recombinase polymerase amplification; RLAMP, rolling circle extension-assisted loop-mediated isothermal amplification; SDA, strand-displacement amplification; ssDNA, single-stranded DNA; ThT, Thioflavin T; vOECT, vertical organic electrochemical transistor.

Note: Analytical performance values are reported in the context used by the original studies and should not be compared directly across platforms because input material, background, amplification, readout, and LOD definitions differ. Methylation-fraction values are therefore described as the lowest reported discriminable fractions under the original study conditions rather than as a standardized minimum detectable methylation fraction. Molecular/specimen evidence (Axis A) and clinical evidence maturity (Axis B) refer to the two-axis framework in main-text Table 3. Axis A reflects proximity to the intended liquid-biopsy specimen rather than overall assay quality; A3 emphasizes endogenous biological authenticity, whereas A4 adds liquid-biopsy matrix realism using spiked or surrogate material. In vitro target or cell experiments, animal-model evidence, and observational patient comparisons are kept distinct; clinical material does not itself establish clinical utility. Null findings and context-dependent responses are retained where relevant. No direct affinity-to-Cas study of 5mC in cancer-associated cfDNA is represented as a core assay row; the affinity-assisted route is discussed in the main text using explicitly labeled non-CRISPR or modified-base precedents. The study-specific material and clinical-design information in the final column provides the rationale for each A/B assignment. "Not demonstrated" indicates no established B-level evidence for the intended cfDNA use; incomplete reporting does not prove an experiment was never conducted.

Supplementary Table S3. Selected clinical cfDNA methylation benchmarks relevant to biomarker-first CRISPR deployment.

Study	Clinical setting and cohort	Methylation approach	Selected reported performance	Validation context	Implication for targeted CRISPR
Church et al. (2014); PRESEPT DOI: 10.1136/gutjnl-2012-304149	Prospective average-risk CRC screening; 7,941 enrolled; main analysis 53 CRC + 1,457 non-CRC.	Plasma methylated SEPT9; bisulfite + commercial PCR.	Sensitivity 48.2%; specificity 91.5%; stage I ≈35%; advanced-adenoma sensitivity 11.2%.	Prospective observational screening validation.	Prospective screening performance can be markedly lower than retrospective case-control estimates; low-copy stochastic sampling remains relevant.
Klein et al. (2021) DOI: 10.1016/j.annonc.2021.05.806	Independent validation of a targeted methylation MCED test; 4,077 participants.	High-dimensional targeted cfDNA methylation classifier.	Specificity 99.5%; overall sensitivity 51.5%; stage I 16.8%, II 40.4%, III 77.0%, IV 90.1%; cancer-signal-origin accuracy 88.7%.	Independent observational diagnostic validation.	Benchmark for specificity and tissue localization; these clinical properties cannot be inferred from analytical LOD.
Mo et al. (2023a); ColonES DOI: 10.1016/j.eclinm.2022.101717	Multicenter CRC/advanced-adenoma cohort; 1,138 individuals.	191 cfDNA methylation-haplotype markers.	Blinded validation: CRC sensitivity 86.6%, advanced adenoma 79.0%, specificity 88.1%; CRC AUC ≈0.937.	Independent multicenter validation.	Supports regional-marker evaluation in the studied CRC/advanced-adenoma cohorts; incremental value must be retested for a compact CRISPR panel.

Study	Clinical setting and cohort	Methylation approach	Selected reported performance	Validation context	Implication for targeted CRISPR
Mo et al. (2023b); ColonAiQ MRD DOI: 10.1001/jamaoncol.2023.0425	Prospective longitudinal stage I-III CRC; 299 patients.	Six-marker methylation qPCR assay.	Preoperative positivity 78.4%; postoperative ctDNA positivity was associated with recurrence (HR ≈17.5); median molecular lead time ≈3.3 months.	Prospective longitudinal observational prognostic evaluation.	Supports research on postoperative monitoring in the studied CRC setting; does not demonstrate benefit from test-guided treatment.
Pedersen et al. (2022) DOI: 10.1002/cncr.34159	CRC recurrence surveillance; reference population 857 and surveillance population 549.	Quantitative methylated BCAT1/IKZF1.	A 0.07% reporting threshold maintained sensitivity (≈63.6%) while increasing specificity to 97.7% and PPV to 81.7%.	Clinical threshold and surveillance validation.	Threshold choice affected performance in the evaluated CRC surveillance populations; these estimates do not transfer automatically to other settings.
Gao et al. (2023); THUNDER DOI: 10.1016/j.annonc.2023.02.010	Development and independent validation across six common cancers.	ELSA-seq targeted cfDNA methylation classifier.	Independent-validation MCDBT-1: sensitivity ≈69.1%, specificity ≈98.9%, predicted-origin accuracy ≈83.2%.	Independent clinical validation.	Evidence benchmark for multi-cancer diagnostic claims; high-dimensional discovery remains complementary to targeted deployment.
Wang et al. (2024); ColonAiQ community screening DOI: 10.1186/s12916-024-03777-2	Average-risk community implementation; 105,285 participants.	Compact multilocus methylation PCR assay.	Test-positive rate 6.42%; among positives completing colonoscopy, CRC PPV 1.92% and advanced-adenoma PPV 13.44%; colonoscopy adherence 48.56%.	Real-world screening implementation, with incomplete verification of negatives.	Real-world performance is shaped by prevalence, PPV, follow-up adherence, and verification bias; sensitivity cannot be inferred without systematic reference testing.
Nguyen et al. (2025); SPOT-MAS DOI: 10.1186/s12916-025-03929-y	Prospective validation in 9,024 asymptomatic adults with 12-month follow-up.	Multimodal targeted/global methylation + fragment size + CNV + end motifs.	Sensitivity 70.83%; specificity 99.71%; PPV 39.53%; tissue-of-origin accuracy 52.94% among true positives.	Prospective observational intended-use validation.	MCED benchmark illustrating why a compact CRISPR assay needs a clearly defined intended use rather than replication of a high-dimensional model.

Study	Clinical setting and cohort	Methylation approach	Selected reported performance	Validation context	Implication for targeted CRISPR
Patel et al. (2026) DOI: 10.1002/cncr.70382	Prediagnostic ACS cohort; 2,754 analyzed, including 1,383 cancers diagnosed within 3 years.	Locked methylation-based MCED classifier in archived prediagnostic plasma.	Signal-detection rate ≈15.2%, 6.0%, and 2.3% at ≈1, 2, and 3 years before diagnosis; non-cancer test-negative rate 100% in this cohort; mean lead time among detected cancers ≈323 days.	Large retrospective prediagnostic validation.	Detection rates varied with time to diagnosis in this prediagnostic cohort; the results do not isolate tumor shedding as the cause or define a fixed sensitivity in other populations.
Nakamura et al. (2026); GALAXY MRD DOI: 10.1038/s41698-026-01277-5	Tissue-agnostic methylation MRD validation in colorectal cancer.	Genome-wide methylome-enrichment MRD assay.	Single postoperative MRD-window sensitivity ≈58.5%; adding serial surveillance increased sensitivity to ≈84.4%.	Longitudinal observational patient validation.	Serial sampling detected additional recurrence-associated signals in this cohort; outcome benefit from test-guided intervention was not established by this comparison.
Zhang et al. (2026); high-risk liver cancer prospective trial DOI: 10.1002/ctm2.70687	Four-center prospective high-risk liver cohort; 1,097 participants.	Three-marker cfDNA methylation model (LC-HMC).	Overall sensitivity 94.43%, specificity 95.16%; stage I sensitivity 93.10%.	Prospective high-risk intended-use validation.	Supports diagnostic evaluation in the studied high-risk liver population; neither average-risk performance nor clinical outcome benefit can be inferred.
Qu et al. (2026) DOI: 10.21037/jgo-2026-0689	Retrospective CRC study; development: 10 CRC/11 healthy; held-out test: 4 CRC/5 healthy; 10 polyps.	MBD-enriched cfDNA sequencing; 12-region model.	Test AUC 0.85 (95% CI 0.579–1.000); sensitivity 75.0% (3/4); specificity 60.0% (3/5).	Small internal held-out test; no external validation.	Candidate affinity-derived markers; polyp discrimination remains uncertain; no Cas readout.

Study	Clinical setting and cohort	Methylation approach	Selected reported performance	Validation context	Implication for targeted CRISPR
Kaur et al. (2026); Asima Rev DOI: 10.34133/csbj.0181	Retrospective: 46 untreated CRC; 33 controls.	Non-CRISPR electrical impedance; aggregation associated with methylation state.	Sensitivity 95.65% (95% CI 85.47–99.23%); specificity 93.94% (95% CI 80.39–98.92%).	Prespecified, geometry-normalized threshold; four samples overlapped with prior cohort.	Global-readout comparator; independent prospective screening evaluation remains necessary.

Abbreviations: ACS, American Cancer Society; AUC, area under the curve; cfDNA, cell-free DNA; CI, confidence interval; CNV, copy-number variation; CRC, colorectal cancer; HR, hazard ratio; MBD, methyl-CpG-binding domain; MCED, multi-cancer early detection; MRD, molecular residual disease; NPV, negative predictive value; PPV, positive predictive value; qPCR, quantitative polymerase chain reaction.

Note: Performance estimates are specific to the cohort and intended use and should not be compared directly across studies with different prevalence, stage distributions, reference standards, or validation designs. Diagnostic and prognostic observational evidence does not establish a causal role for the marker or benefit from test-guided intervention. Reported point estimates retain the uncertainty of the original study; lack of an interval in this summary does not imply a precise or transferable estimate.

Supplementary Table S4. Practical checklist for biomarker-first CRISPR methylation biosensor deployability.

Dimension	Key question	Minimum supporting evidence or measurement	Common failure mode
Clinical relevance	Does the methylation feature address a defined clinical use case?	Effect size and performance in the intended patient groups; explicitly defined use case (e.g., screening, triage/high-risk surveillance, or MRD/monitoring), comparator, endpoint, acceptable false-positive burden, and turnaround requirements. For longitudinal monitoring, evidence that the selected feature remains trackable across treatment and disease evolution should also be considered.	Selecting a locus because it is technically convenient rather than clinically informative, or assuming that a baseline-positive marker remains stable during longitudinal monitoring.
Biological specificity	Is the methylation state uncommon in relevant non-cancer backgrounds?	Healthy plasma plus intended-use-relevant benign, inflammatory, and tissue-specific controls.	Excellent molecular discrimination of a biologically nonspecific marker.
cfDNA recoverability	Is the informative region consistently present on recoverable plasma fragments?	Empirical locus coverage, fragment-length/end distributions, and expected target copies in the intended population.	Choosing a strong tissue marker that is rarely recovered intact from plasma.

Dimension	Key question	Minimum supporting evidence or measurement	Common failure mode
CRISPR accessibility	Can the validated region be interrogated with acceptable guide, PAM, enzyme-site, and fragment geometry?	Guide activity, PAM availability or engineered alternatives, CpG position/strand, and auxiliary-site compatibility.	Substituting a weaker biomarker solely because the preferred locus has inconvenient geometry.
Quantifiability	Can signal be mapped reproducibly to methylation fraction or molecule count?	Calibration curve; limit of blank, detection, and quantification as appropriate; dynamic range; precision; internal controls; and concordance with an orthogonal methylation method (e.g., qMSP, dPCR, or targeted methylation sequencing) in representative biological samples.	Treating qualitative signal gain as accurate methylation quantification.
Workflow complexity	Can the complete sample-to-answer process preserve molecules and remain robust?	End-to-end recovery, step count, hands-on time, contamination controls, automation potential, matrix robustness, throughput, and turnaround matched to the intended use.	Adding amplification or transduction layers that improve nominal LOD but increase loss, leakage, or variability.
Device and manufacturing readiness	Can the assay be implemented reproducibly in a portable, closed, or scalable biosensor format?	Device architecture; reader requirements; cartridge or fluidic integration; reagent storage and stabilization; fabrication tolerances; surface fouling or drift where relevant; lot-to-lot reproducibility; user steps; power, optical, or electronic requirements; and manufacturability at the intended scale.	Strong benchtop analytical performance without a reproducible route to integrated device fabrication or sample-to-answer operation.
Evidence position	What molecular/specimen evidence and clinical evidence maturity have actually been reached?	Separate assignment on the main-text two-axis framework: Axis A (A1-A5) for molecular/specimen evidence and Axis B (B1-B5) for clinical evidence maturity, with cohort design and assay-locking status stated explicitly.	Treating a realistic specimen, a small patient cohort, or a strong analytical signal as equivalent to independent clinical validity, intended-use performance, or clinical utility.
Minimum analytical reporting	Can another laboratory reconstruct the input and analytical performance experiment?	Plasma volume; total extracted cfDNA mass; reaction DNA mass, sample volume and total volume; replicate allocation; end-to-end recovery and definition; fragment-matched standards; limits of blank, detection and quantification when applicable; lowest discriminable methylation fraction at stated total input; within-run/run-to-run precision; interference and invalid-result criteria.	A nominal concentration or fluorescence difference without a defined molecular budget, calibration or calling rule.

Abbreviations: cfDNA, cell-free DNA; dPCR, digital polymerase chain reaction; CRISPR, clustered regularly interspaced short palindromic repeats; PAM, protospacer-adjacent motif; qMSP, quantitative methylation-specific polymerase chain reaction.
